# Supplementary material for: Revisiting the NPcis mouse model: A new tool to model plexiform neurofibroma
Source: PLoS One. 2024 Jun 20;19(6):e0301040. doi: 10.1371/journal.pone.0301040 (PMC11189233; doi:10.1371/journal.pone.0301040)

S1 Fig

S100, desmin, SMA IHC. Spontaneous sarcoma from NPcis

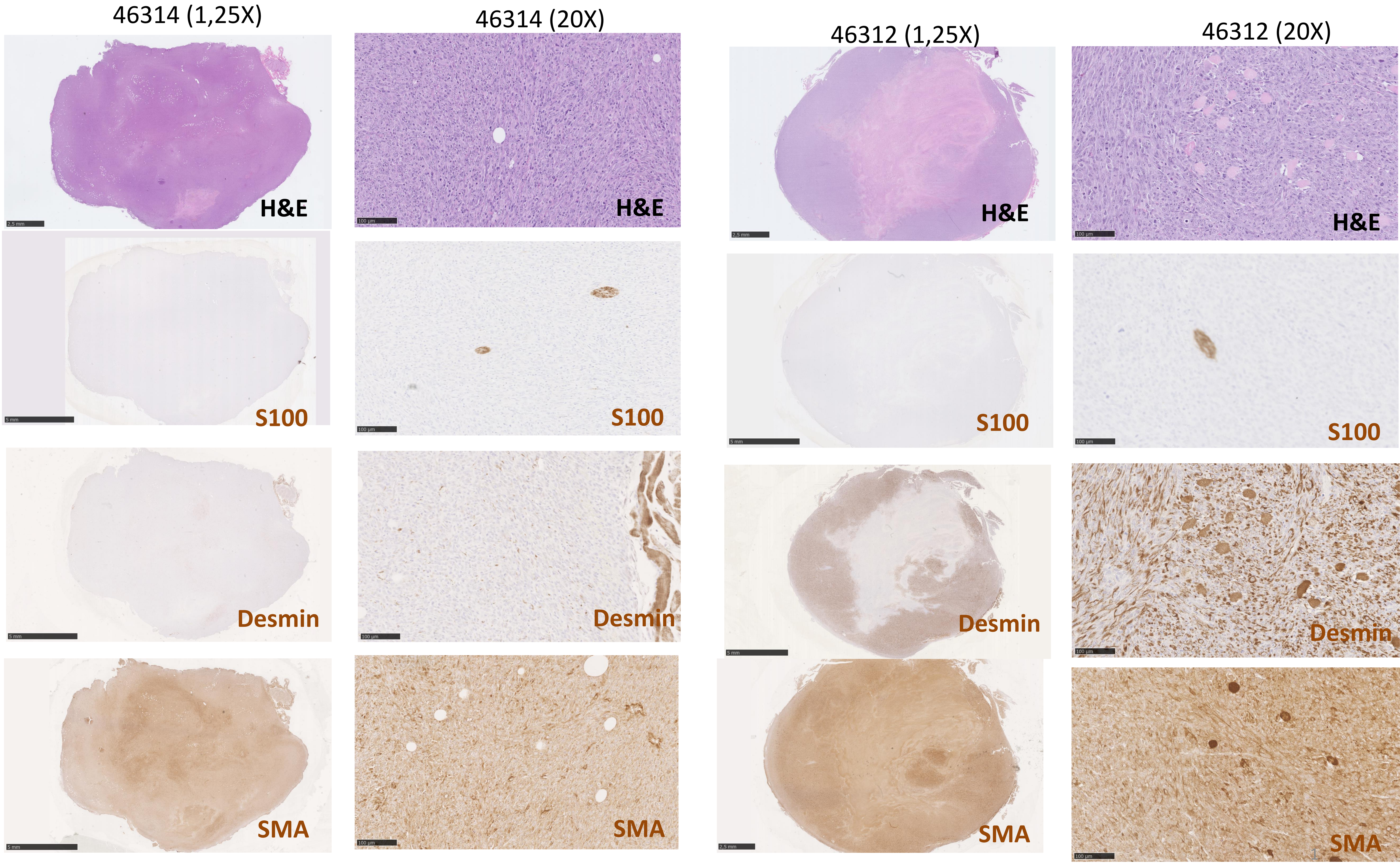

S1 Fig

S100, desmin, SMA IHC. Spontaneous sarcoma from NPcis

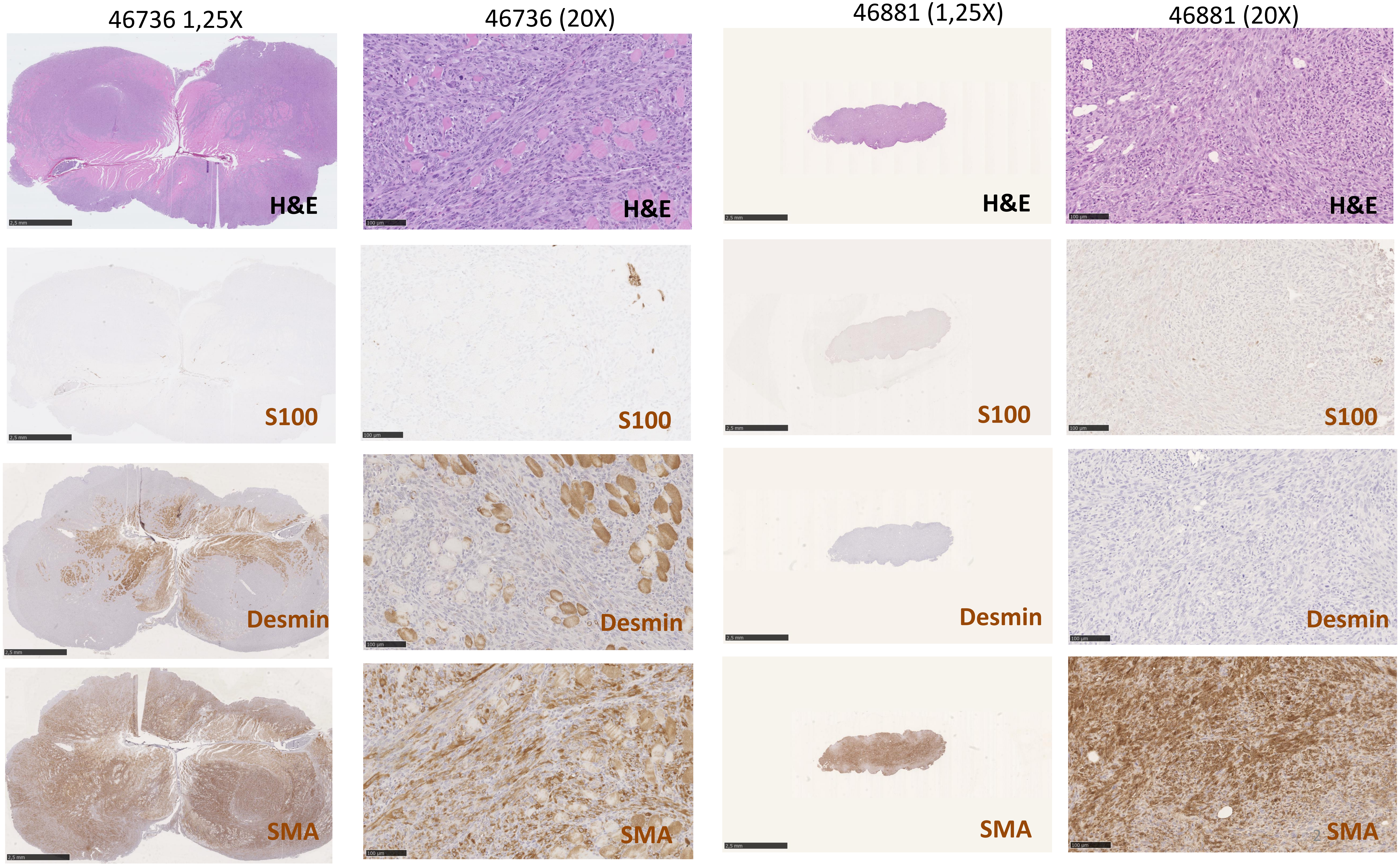

S1 Fig

S100, desmin, SMA IHC. Spontaneous sarcoma from NPcis

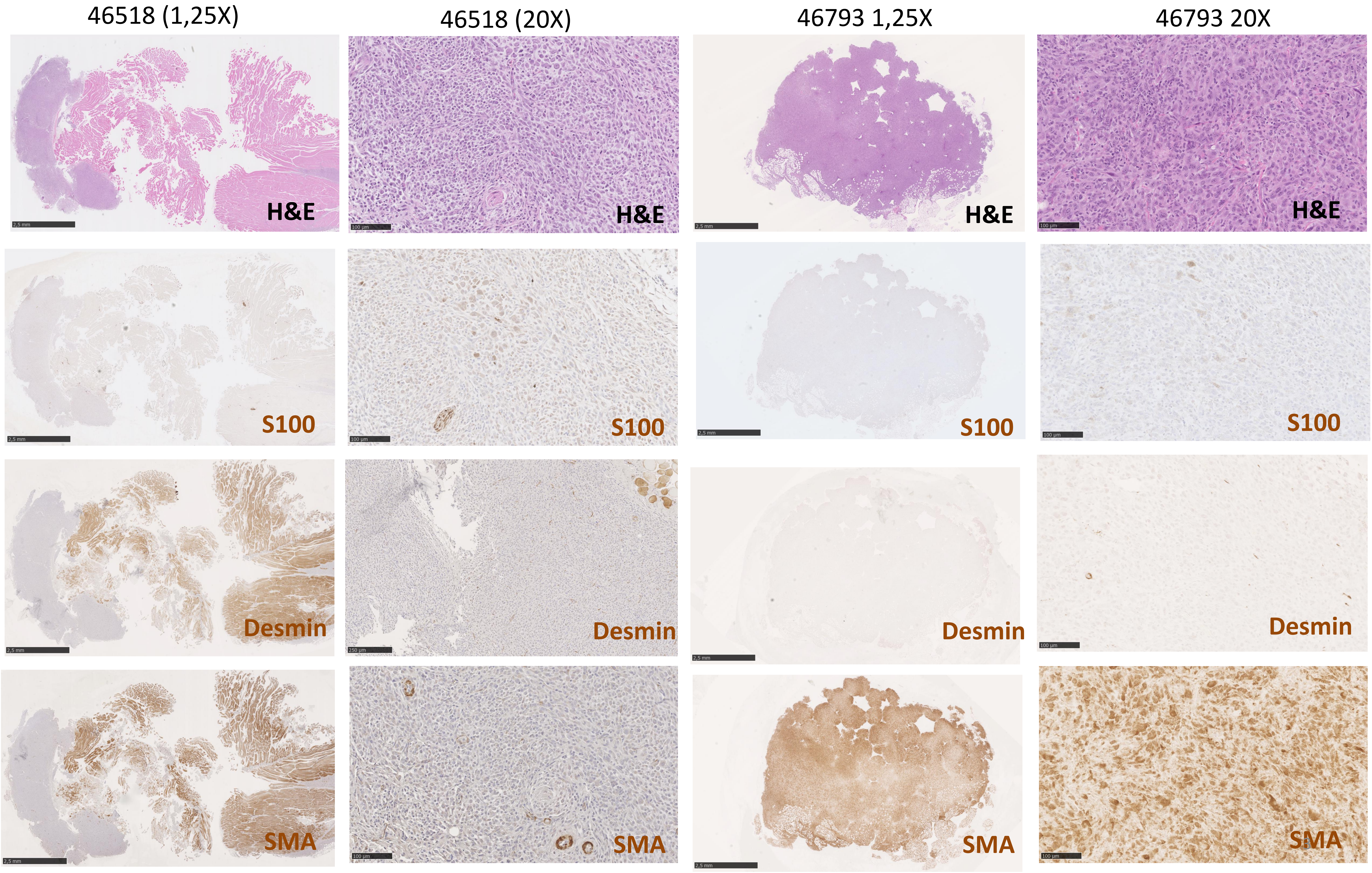

S1 Fig

S100, desmin, SMA IHC. Spontaneous sarcoma from NPcis

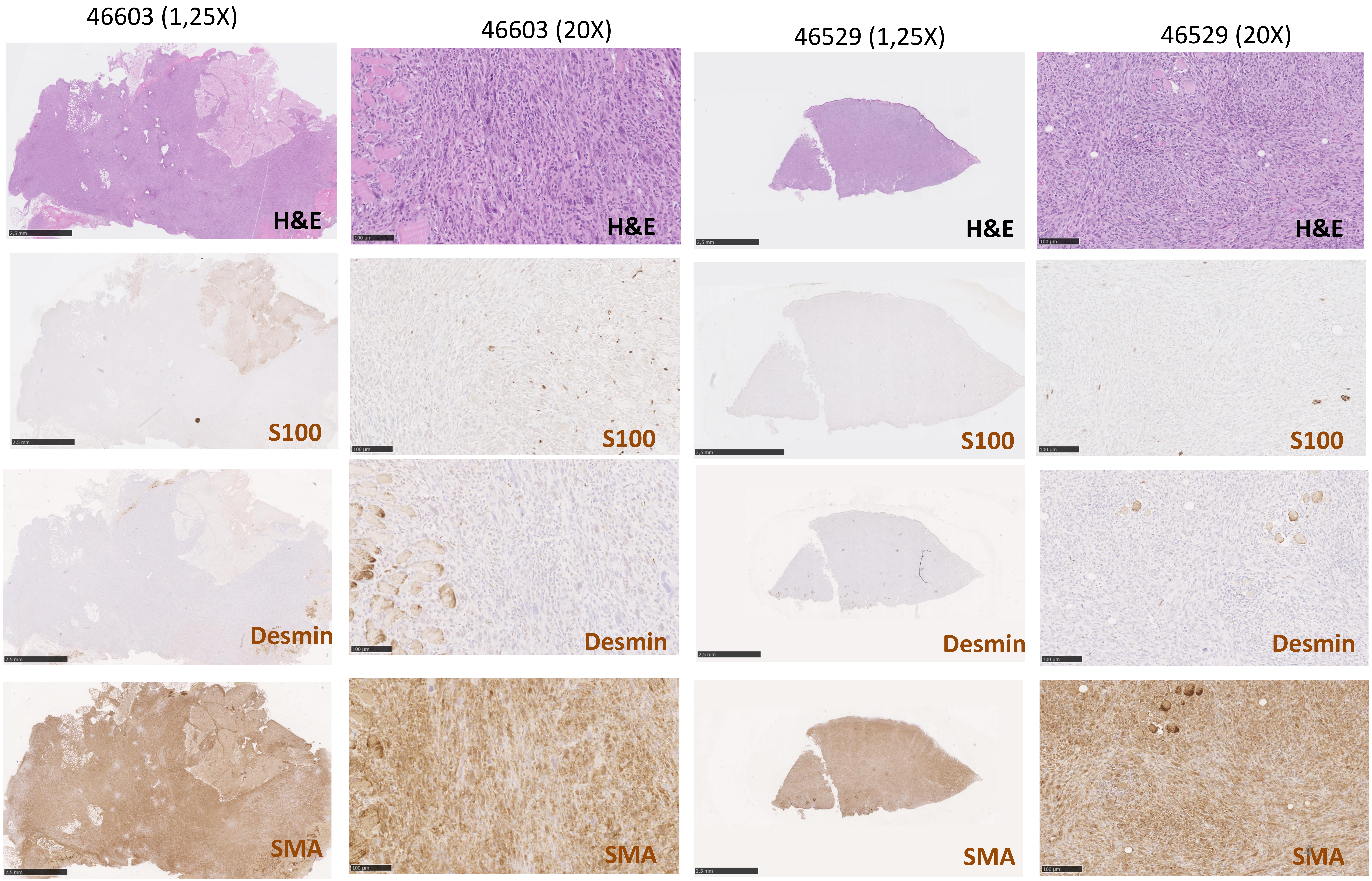

S1 Fig

S100, desmin, SMA IHC. Spontaneous sarcoma from NPcis

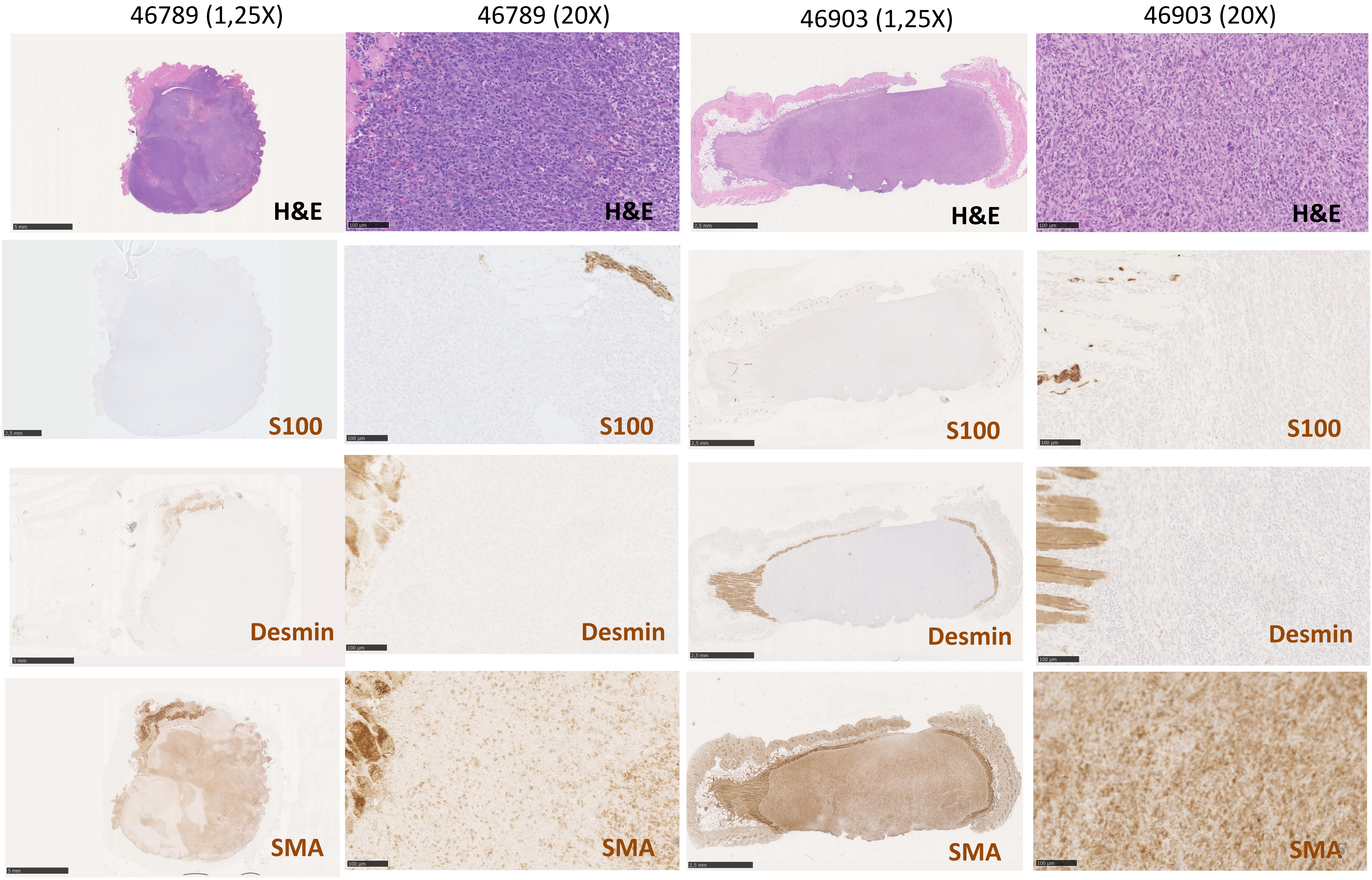

S1 Fig

S100, desmin, SMA IHC. Spontaneous sarcoma from NPcis

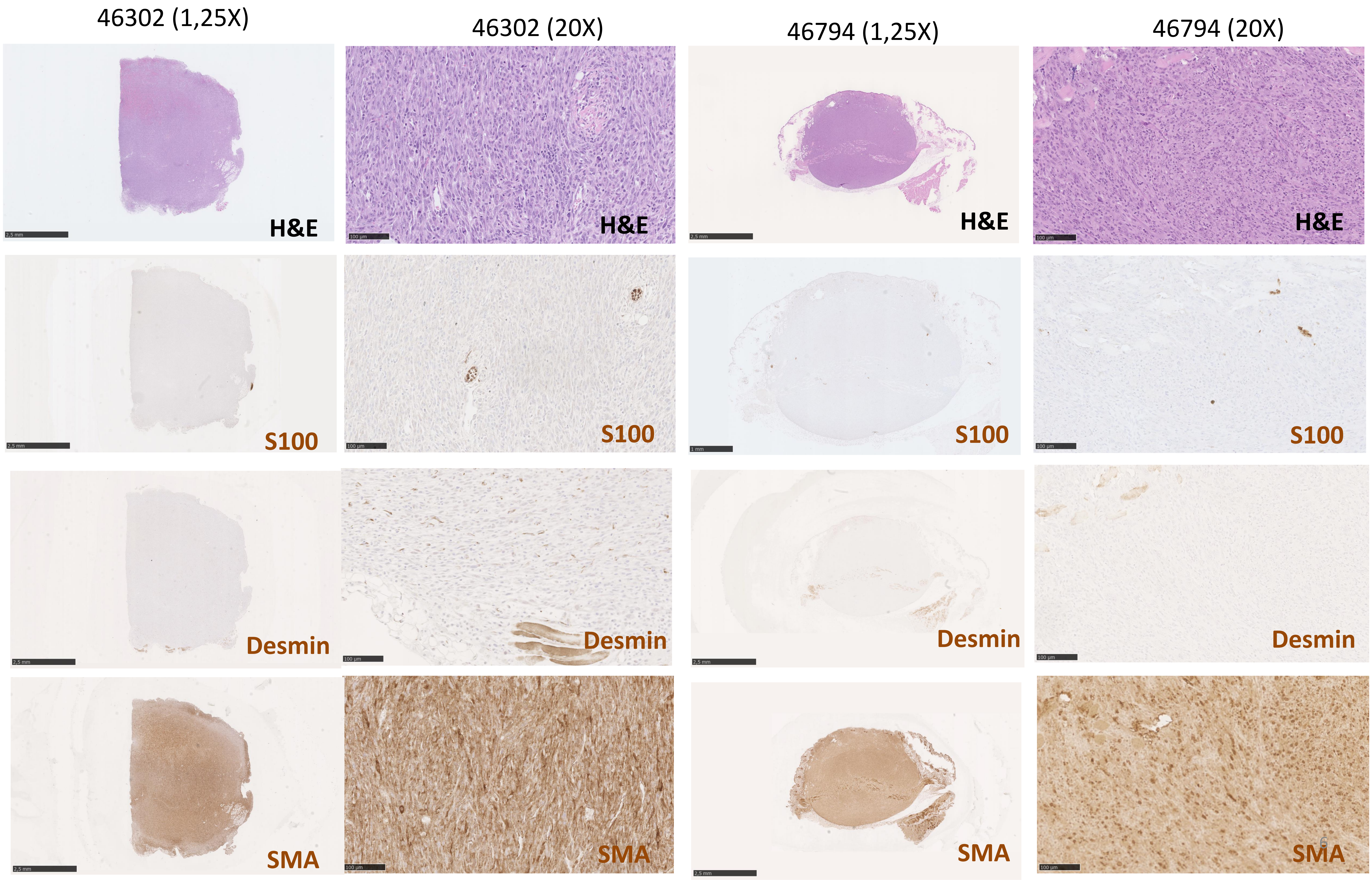

S1 Fig

S100, desmin, SMA IHC. Spontaneous sarcoma from NPcis

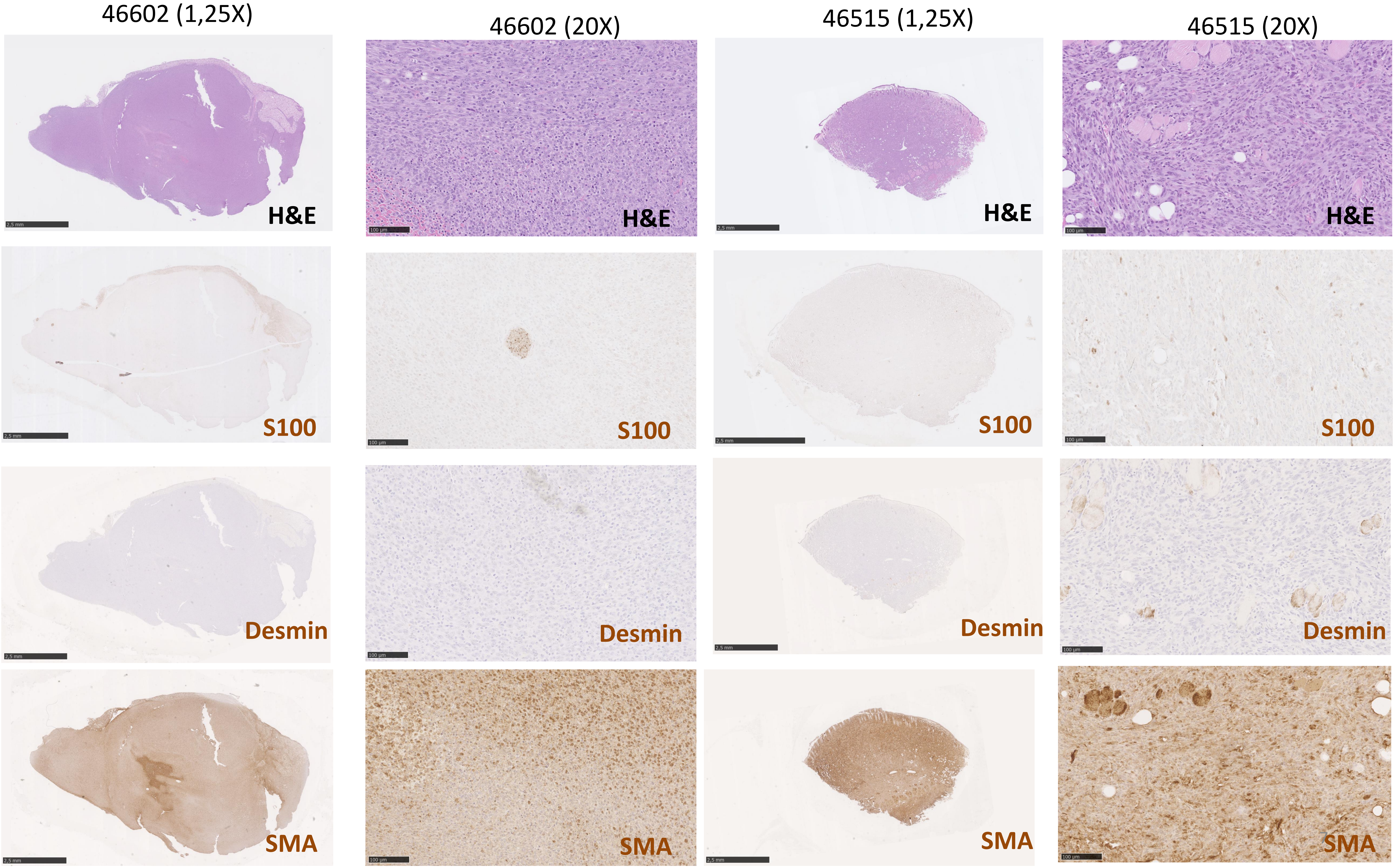

S1 Fig

S100, desmin, SMA IHC. Spontaneous sarcoma from NPcis

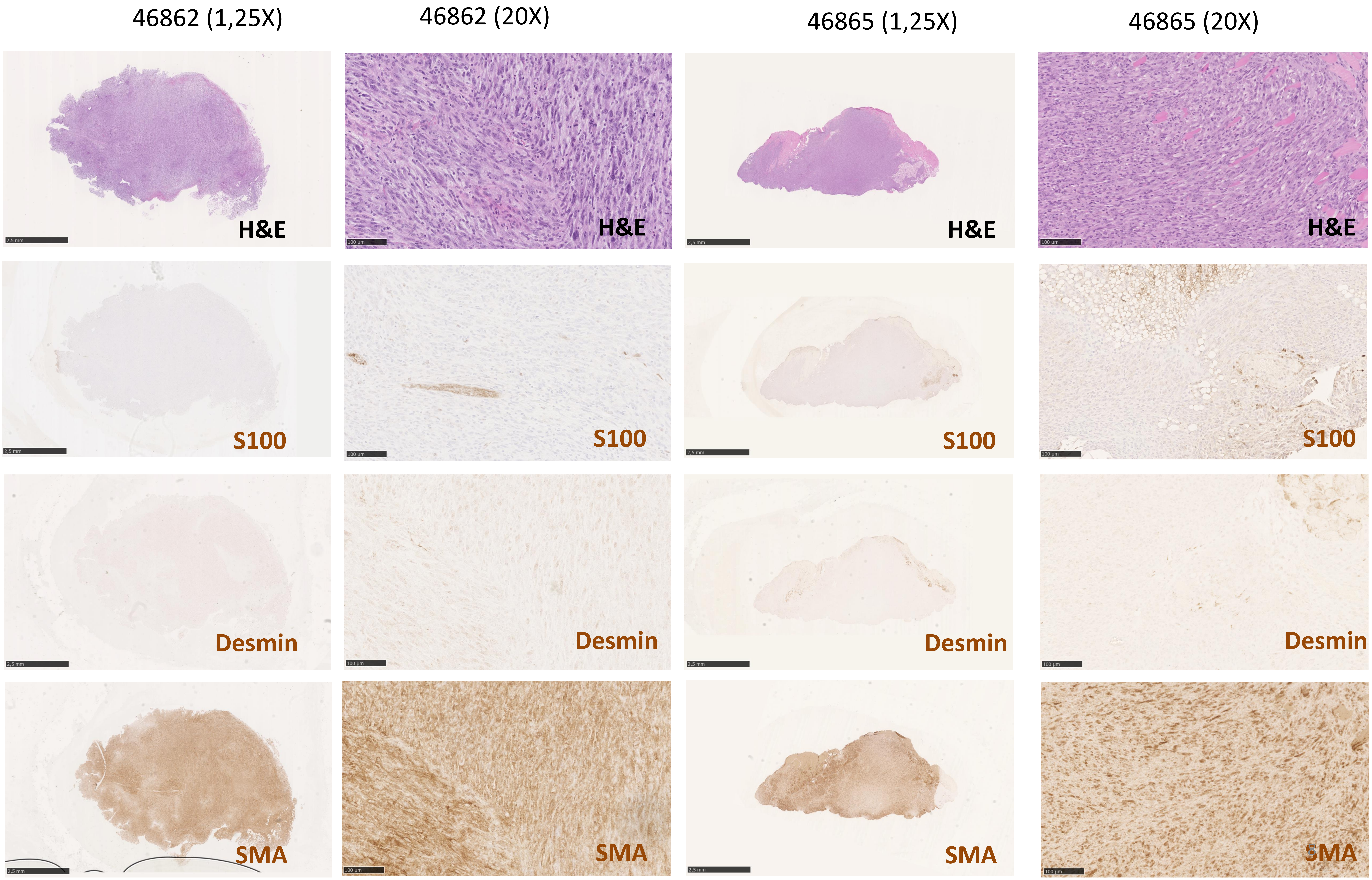

S1 Fig

S100, desmin, SMA IHC. Spontaneous sarcoma from NPcis

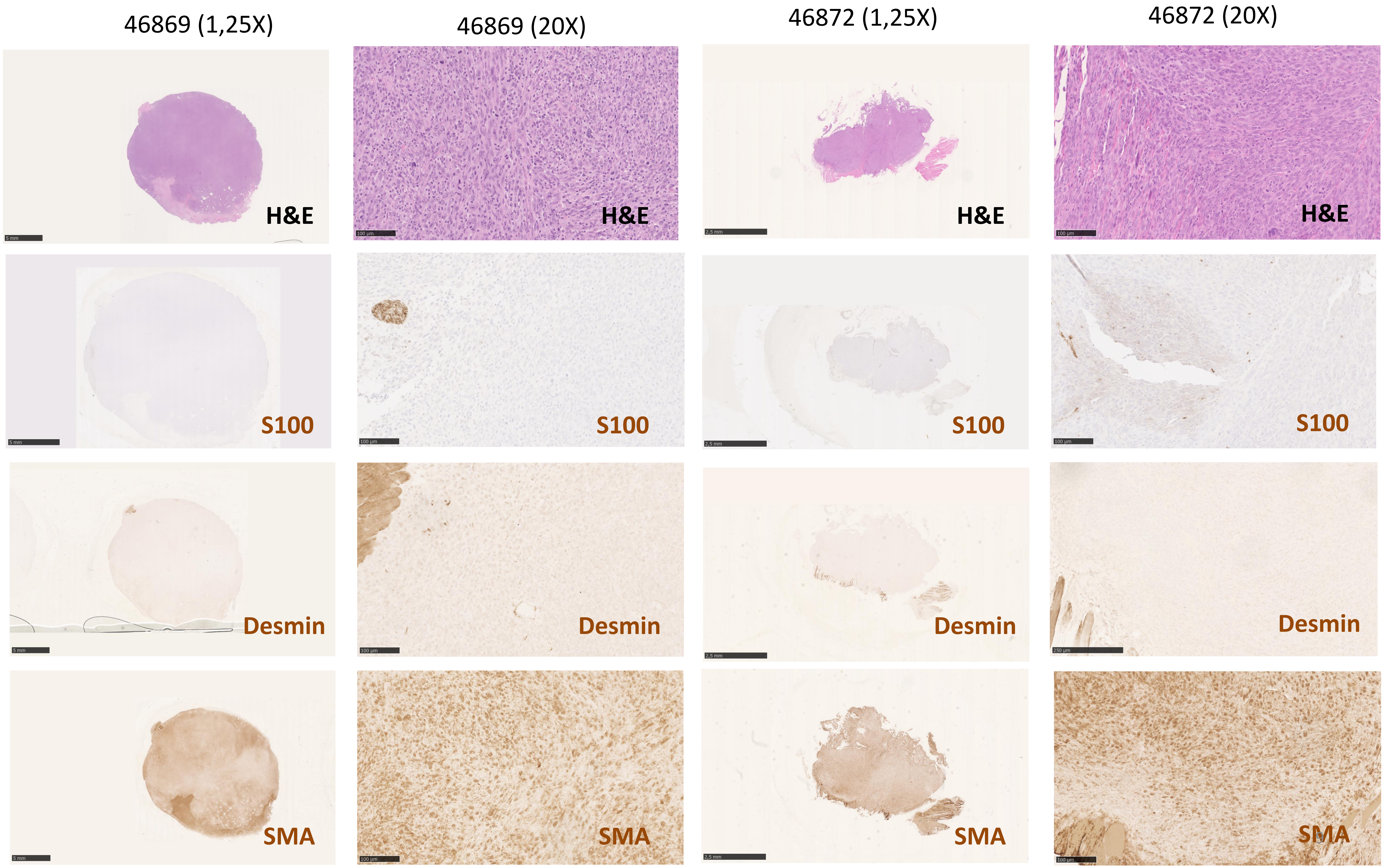

S1 Fig

S100, desmin, SMA IHC. Spontaneous sarcoma from NPcis

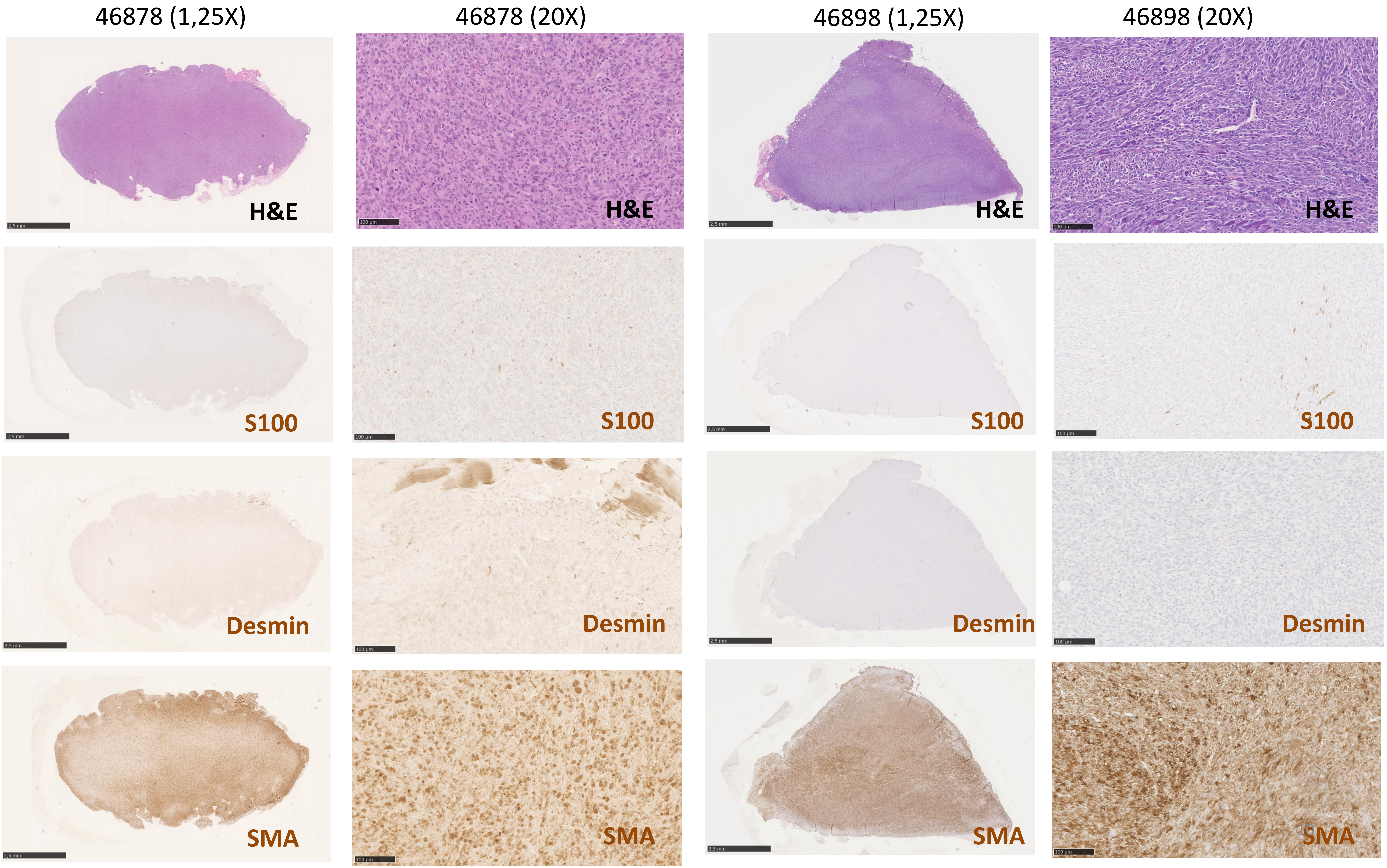

S1 Fig

S100, desmin, SMA IHC. Spontaneous sarcoma from NPcis

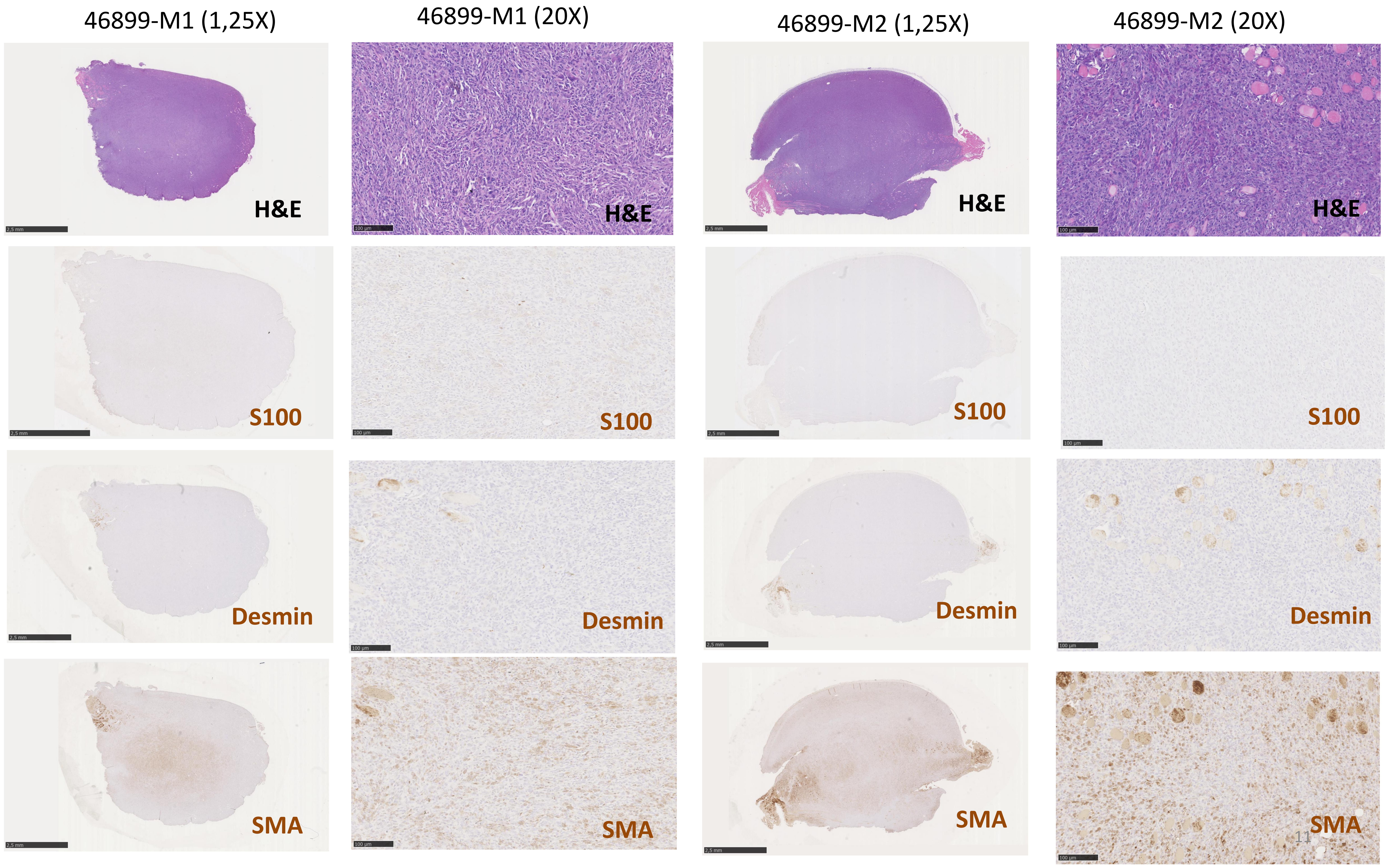

S1 Fig

S100, desmin, SMA IHC. Spontaneous sarcoma from NPcis

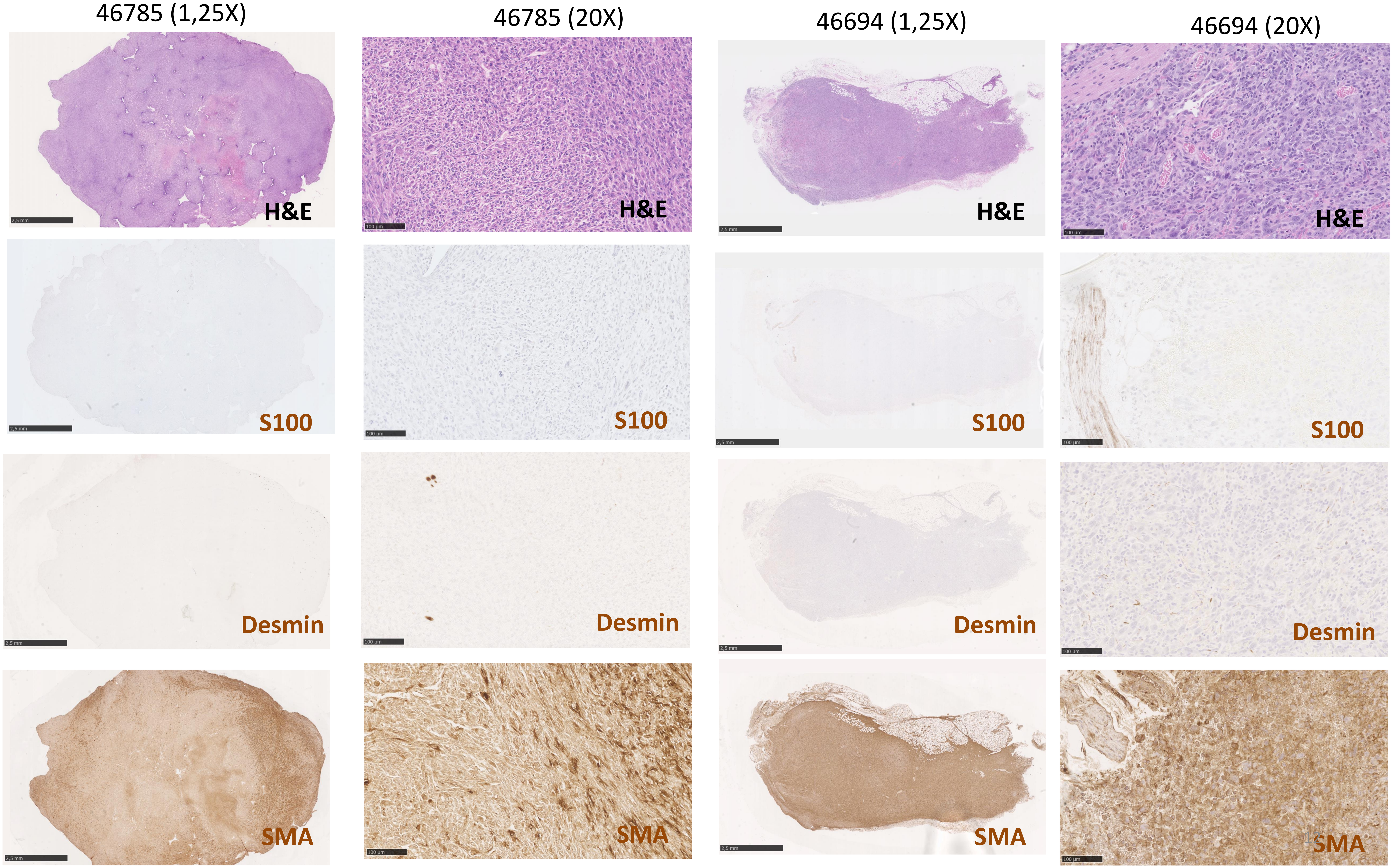

S1 Fig

S100, desmin, SMA IHC. Spontaneous sarcoma from NPcis

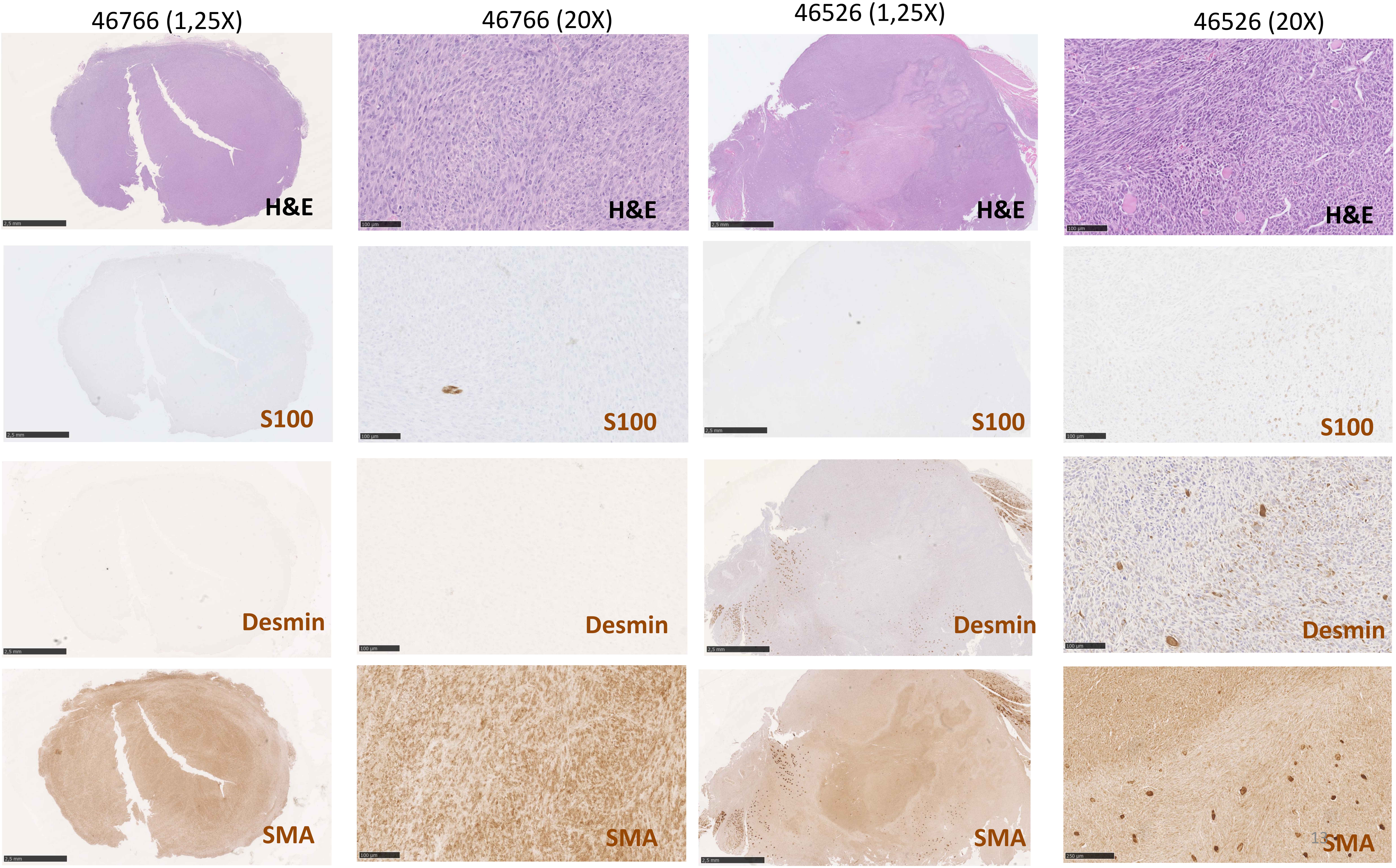

Supplement: S1 Fig — Full histological characterization (H&E and S100, desmin and SMA immunostaining) of sarcoma from NPcis mouse model. (PDF) [file pone.0301040.s001.pdf]
