# Supplementary material for: Revisiting the NPcis mouse model: A new tool to model plexiform neurofibroma
Source: PLoS One. 2024 Jun 20;19(6):e0301040. doi: 10.1371/journal.pone.0301040 (PMC11189233; doi:10.1371/journal.pone.0301040)

S3 Fig

H&E. Injury-induced NPcis sciatic nerves that didn`t develop pNF (needle method)

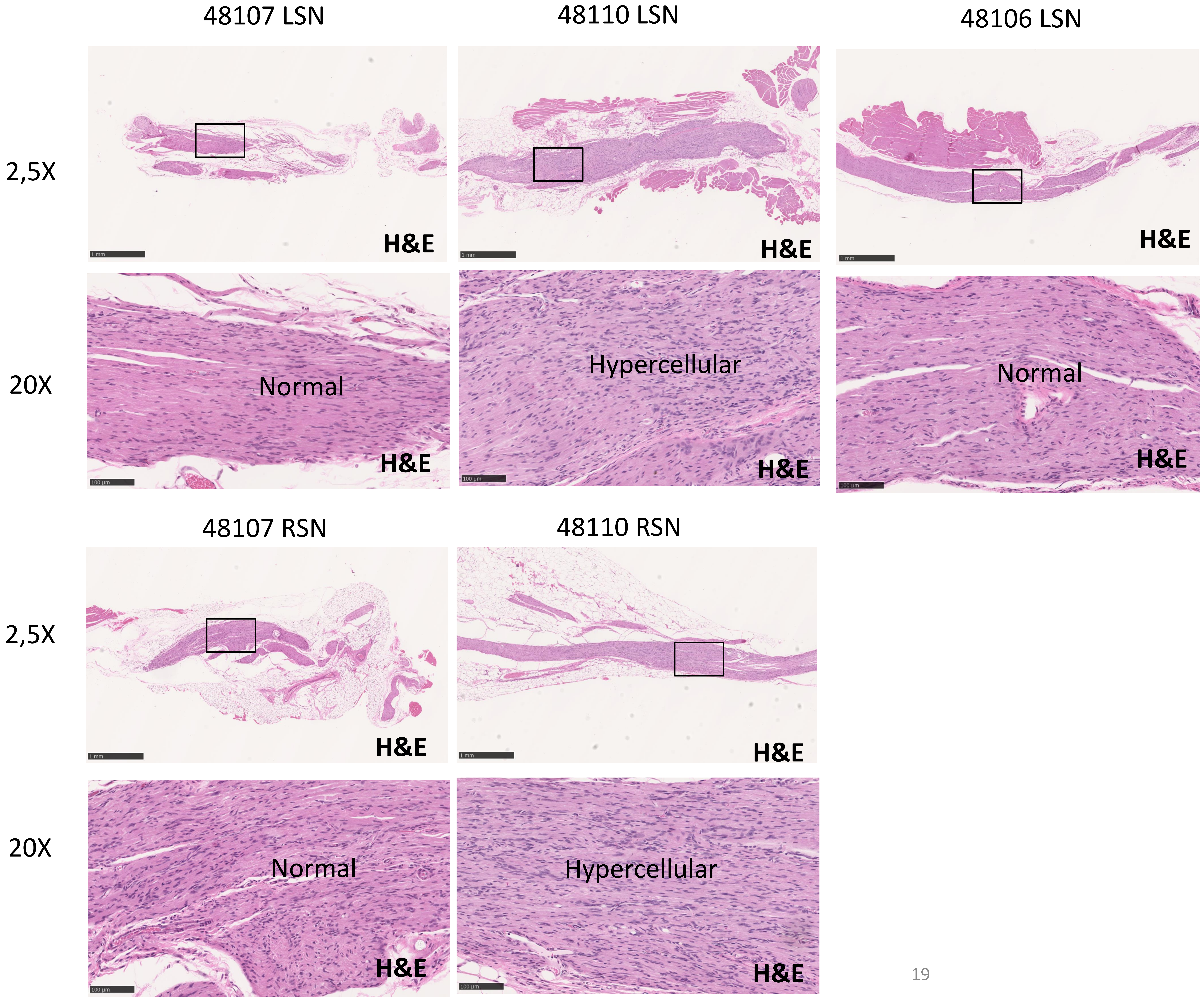

S3 Fig

H&E. Injury-induced NPcis sciatic nerves developing pNF (needle method)

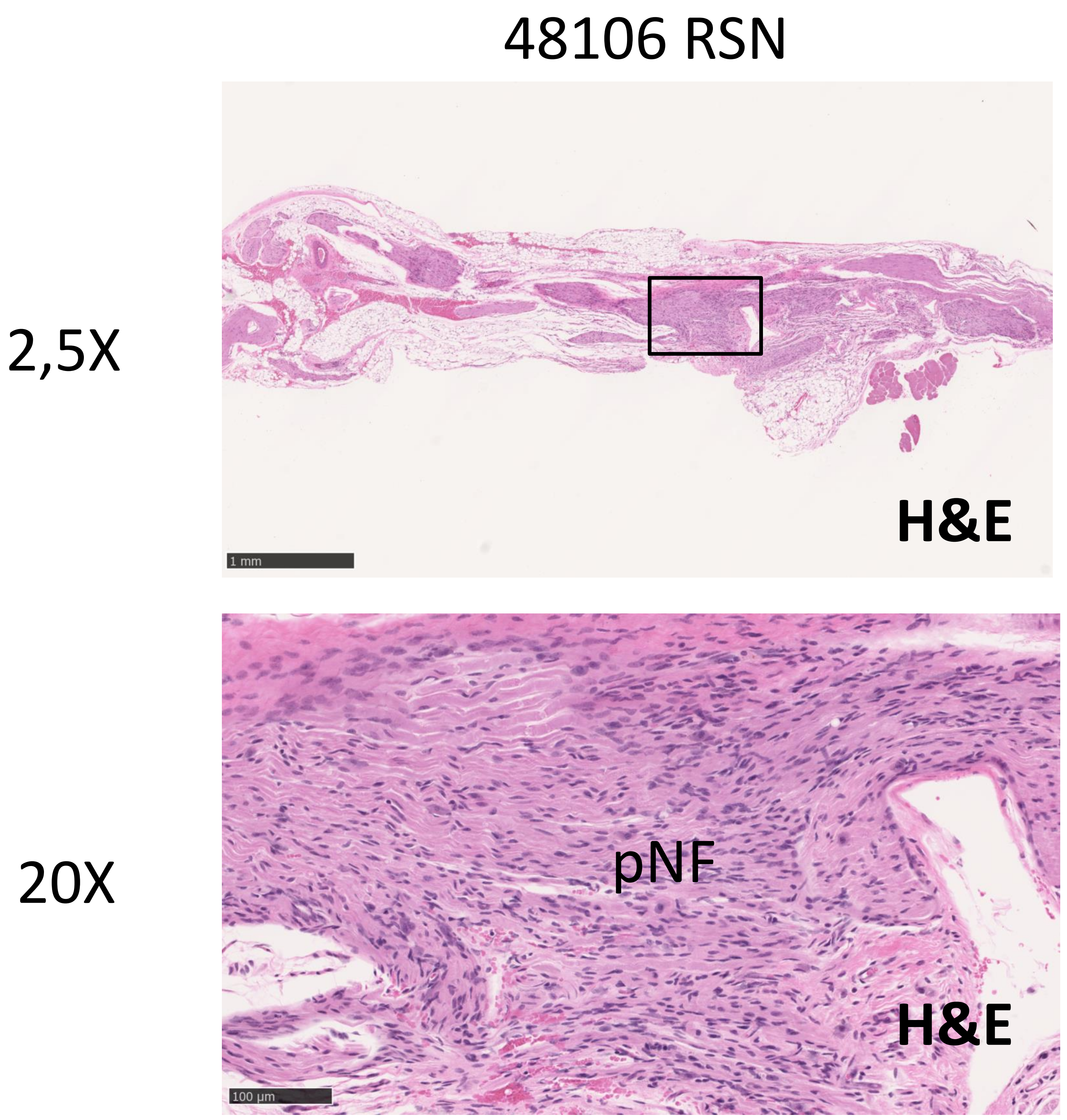

S3 Fig

H&E. Injury-induced NPcis sciatic nerves that didn`t develop pNF (cut method)

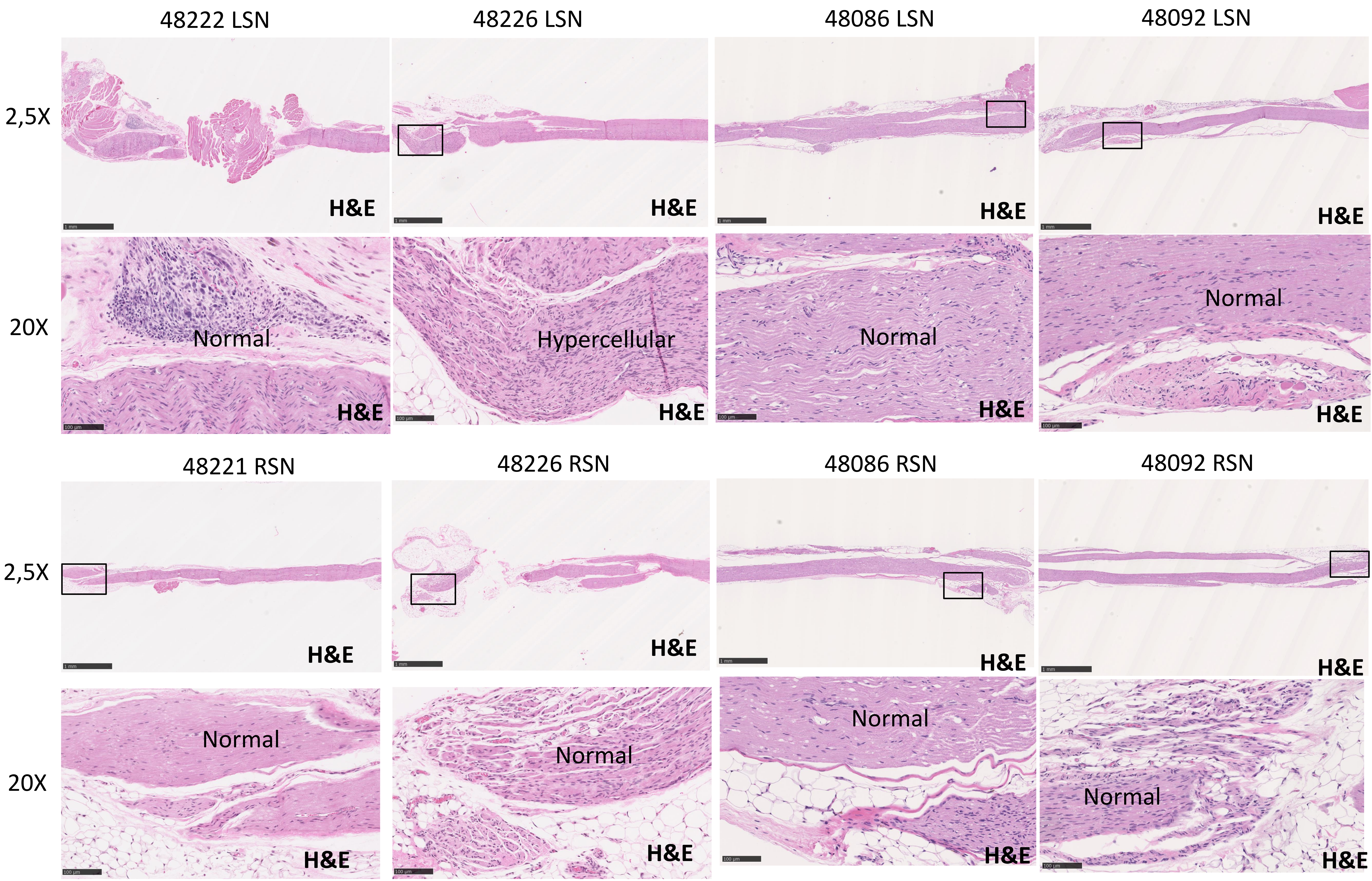

S3 Fig

H&E. Injury-induced NPcis sciatic nerves developing pNF (cut method)

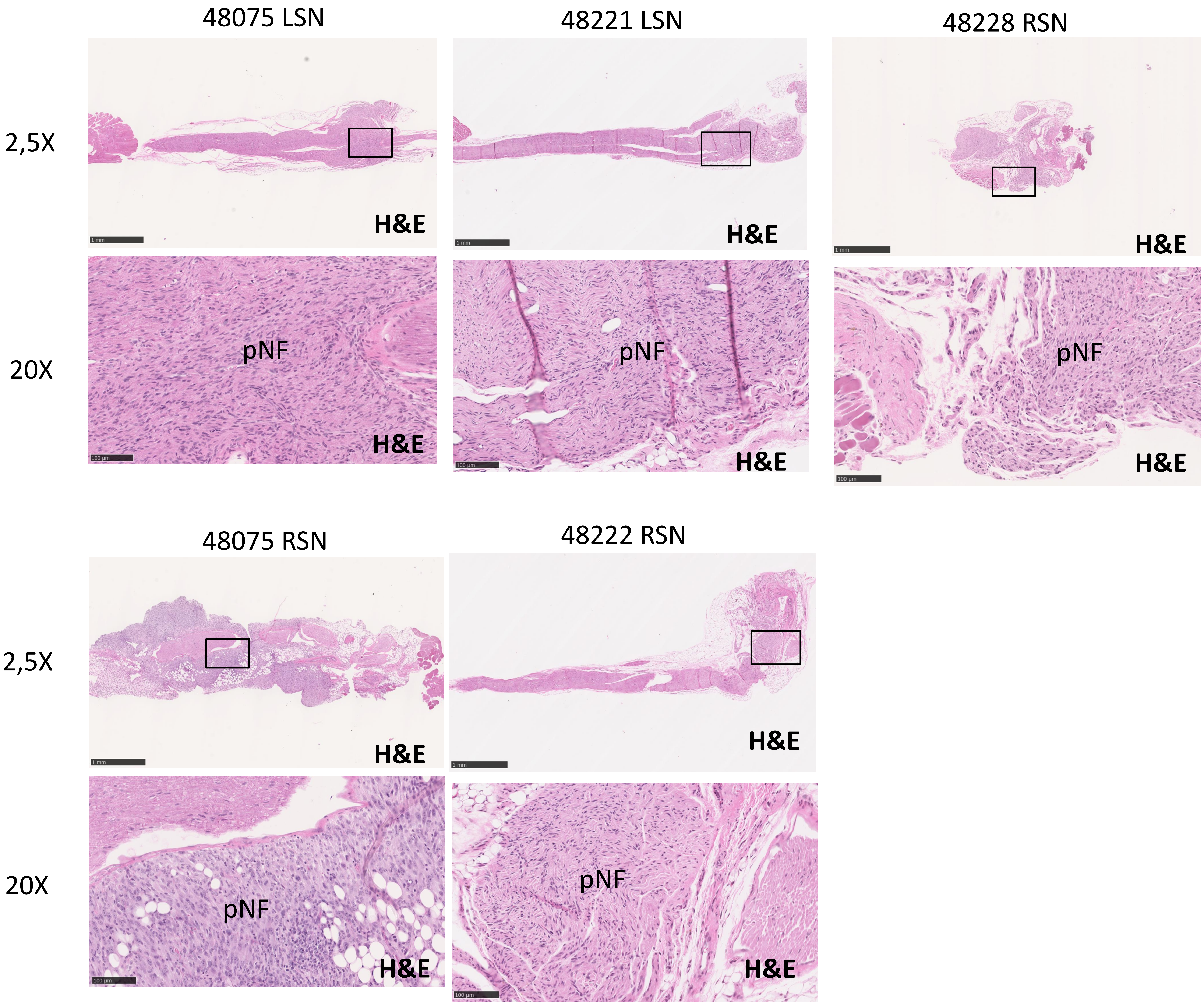

S3 Fig

H&E. Injury-induced NPcis sciatic nerves developing pNF (cut method)

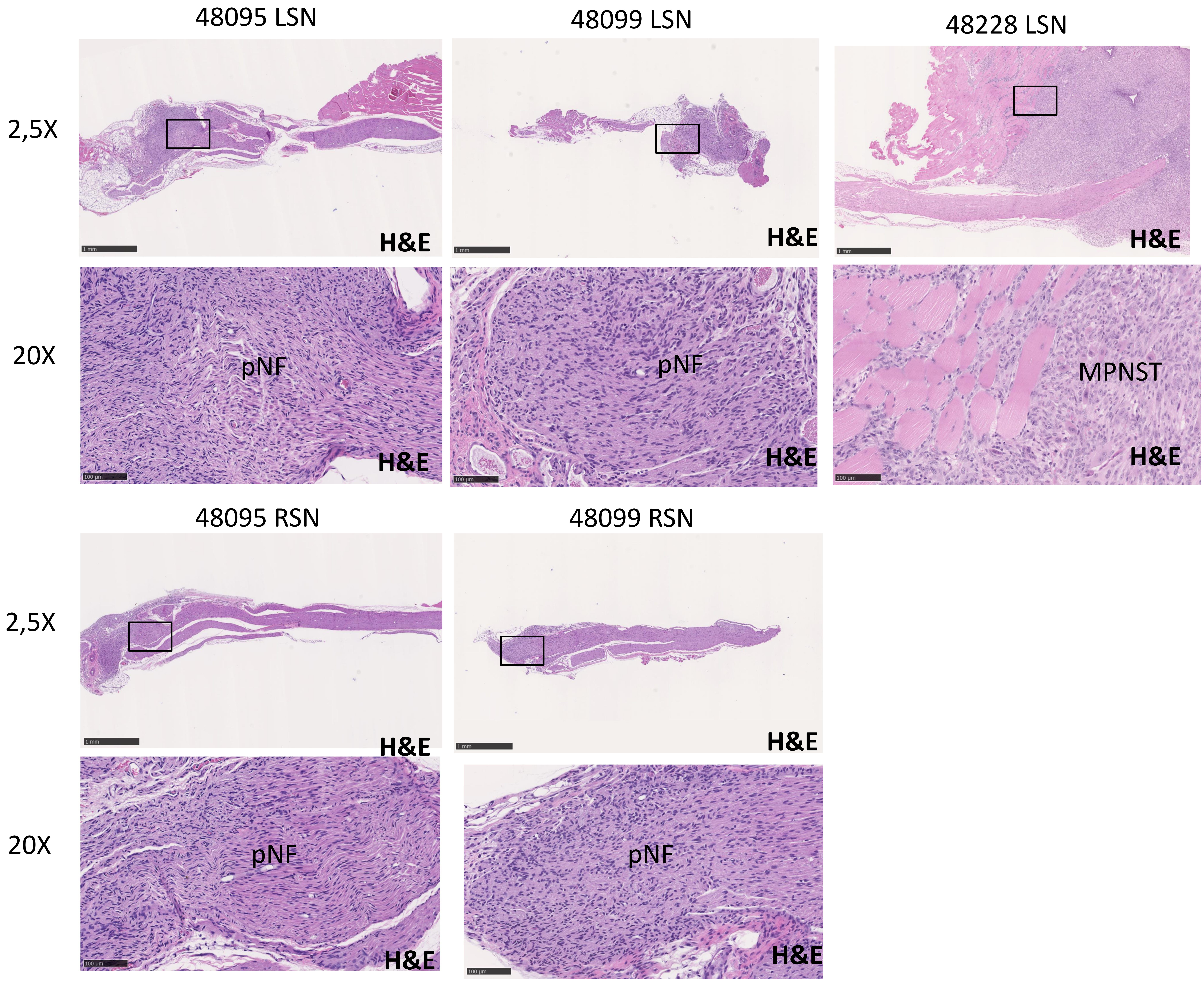

Supplement: S3 Fig — Full histological characterization (H&E) of injury-induced sciatic nerve from the NPcis mouse model comparing the needle and the cut method. (PDF) [file pone.0301040.s003.pdf]
