# Supplementary material for: Revisiting the NPcis mouse model: A new tool to model plexiform neurofibroma
Source: PLoS One. 2024 Jun 20;19(6):e0301040. doi: 10.1371/journal.pone.0301040 (PMC11189233; doi:10.1371/journal.pone.0301040)

S6 Fig

Nf1 and p53 IHC. Injury-induced NPcis sciatic nerves that didn`t develop pNF (needle method)

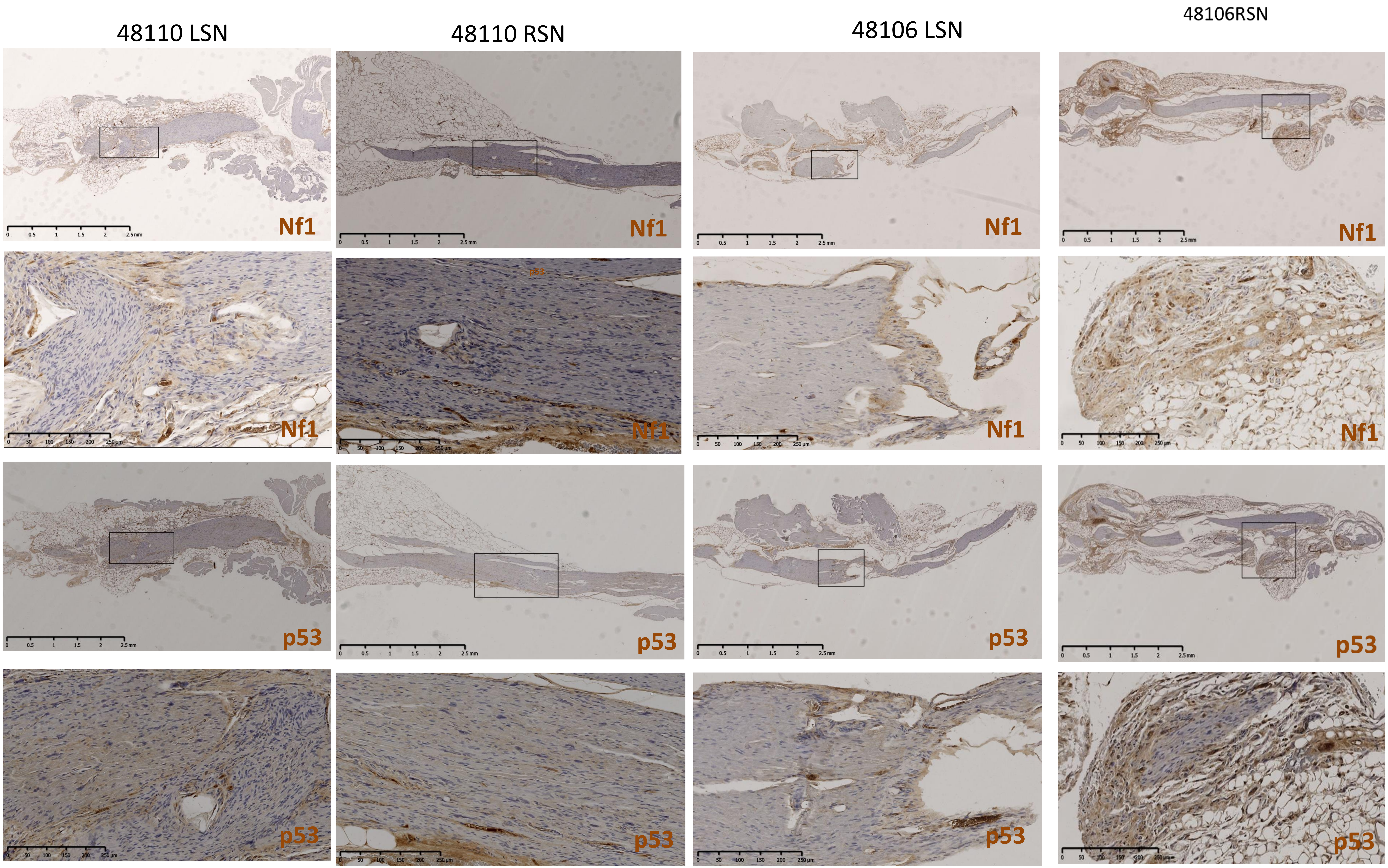

S6 Fig

Nf1 and p53 IHC. Injury-induced NPcis sciatic nerves that didn`t develop pNF (cut method)

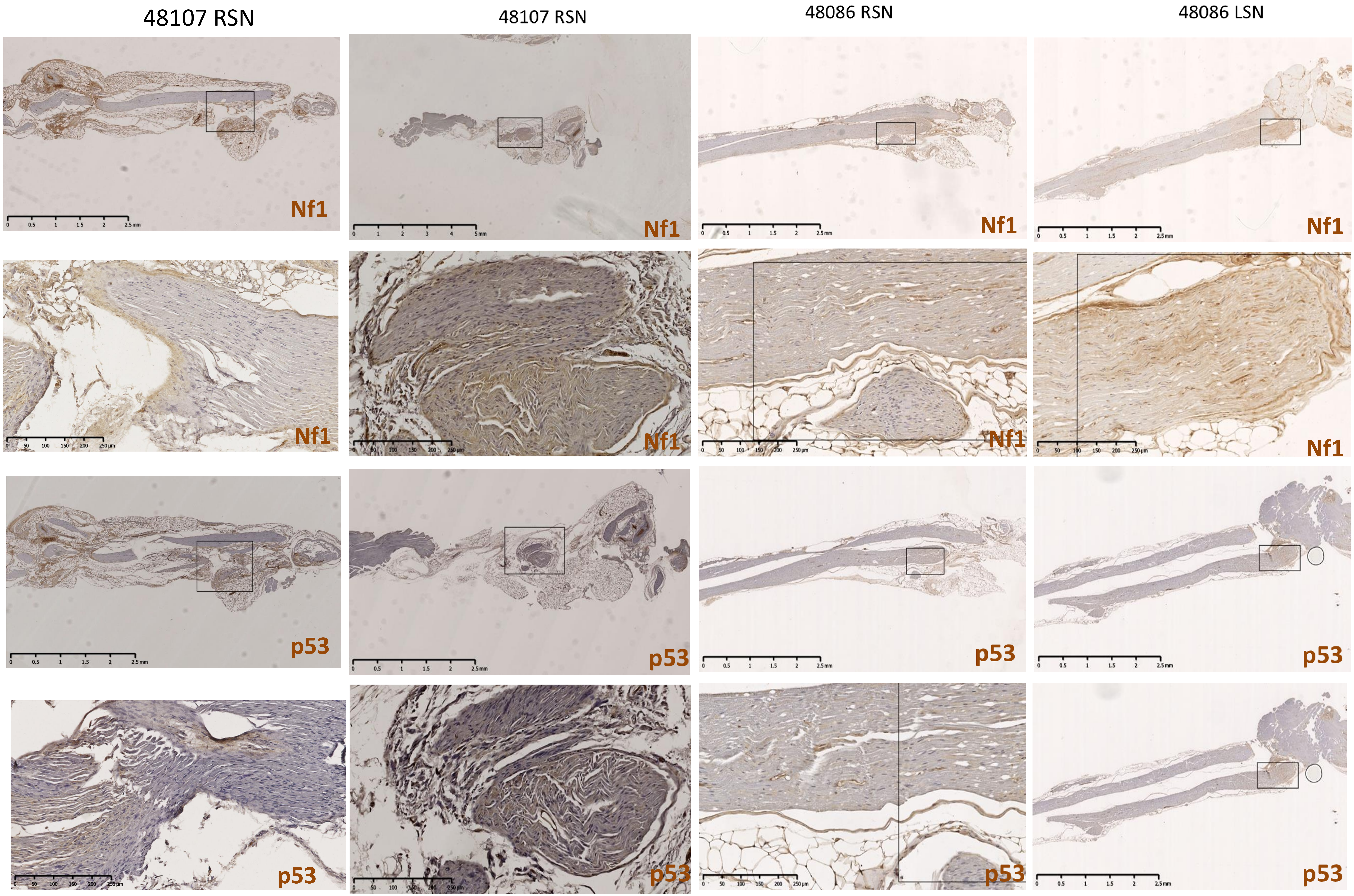

S6 Fig

Nf1 and p53 IHC. Injury-induced NPcis sciatic nerves that didn`t develop pNF (cut method)

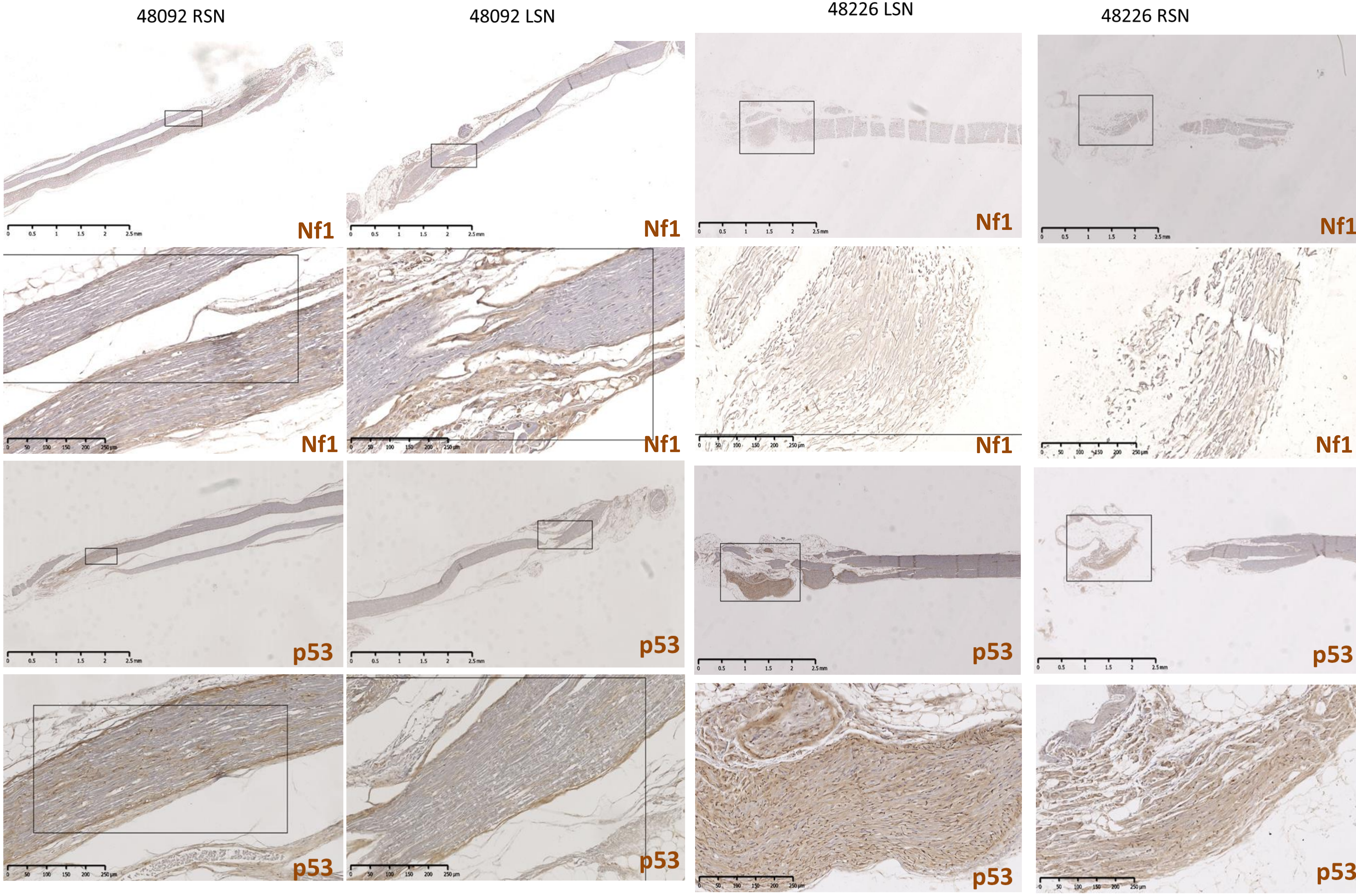

S6 Fig

Nf1 and p53 IHC. Injury-induced NPcis sciatic nerves developing pNF (cut method)

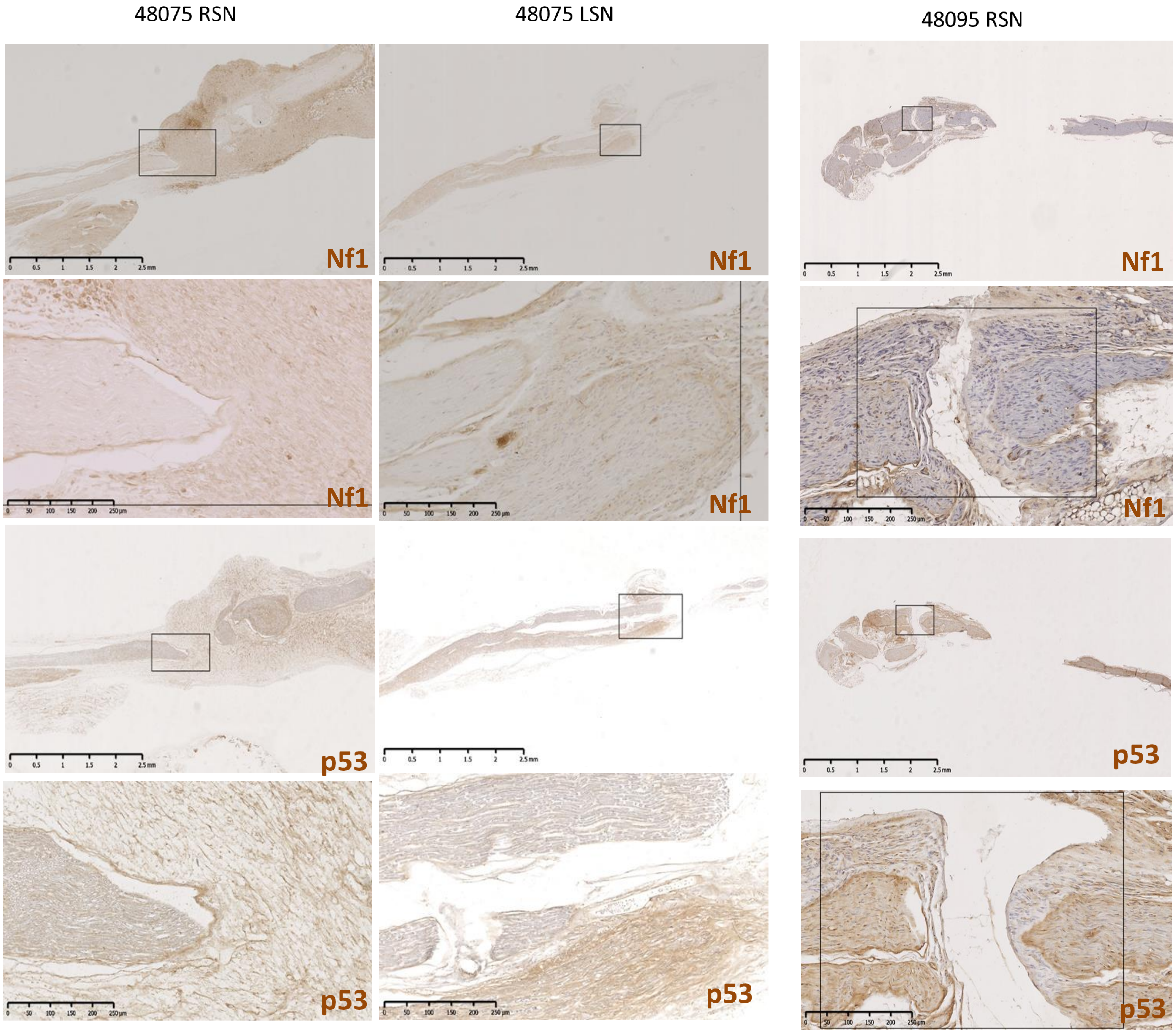

S6 Fig

Nf1 and p53 IHC. Injury-induced NPcis sciatic nerves developing pNF (cut method)

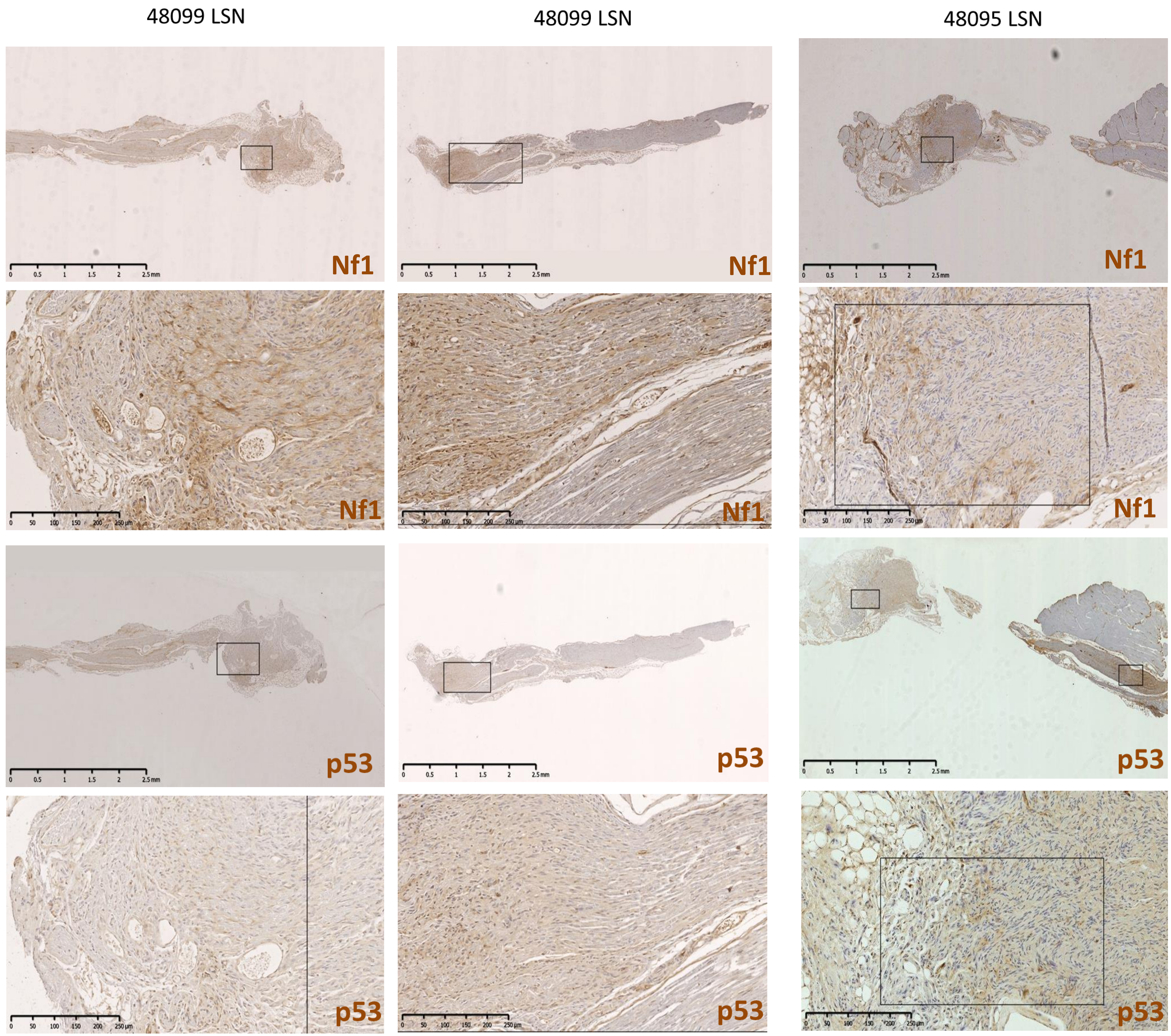

S6 Fig

Nf1 and p53 IHC. Injury-induced NPcis sciatic nerves developing pNF (cut method)

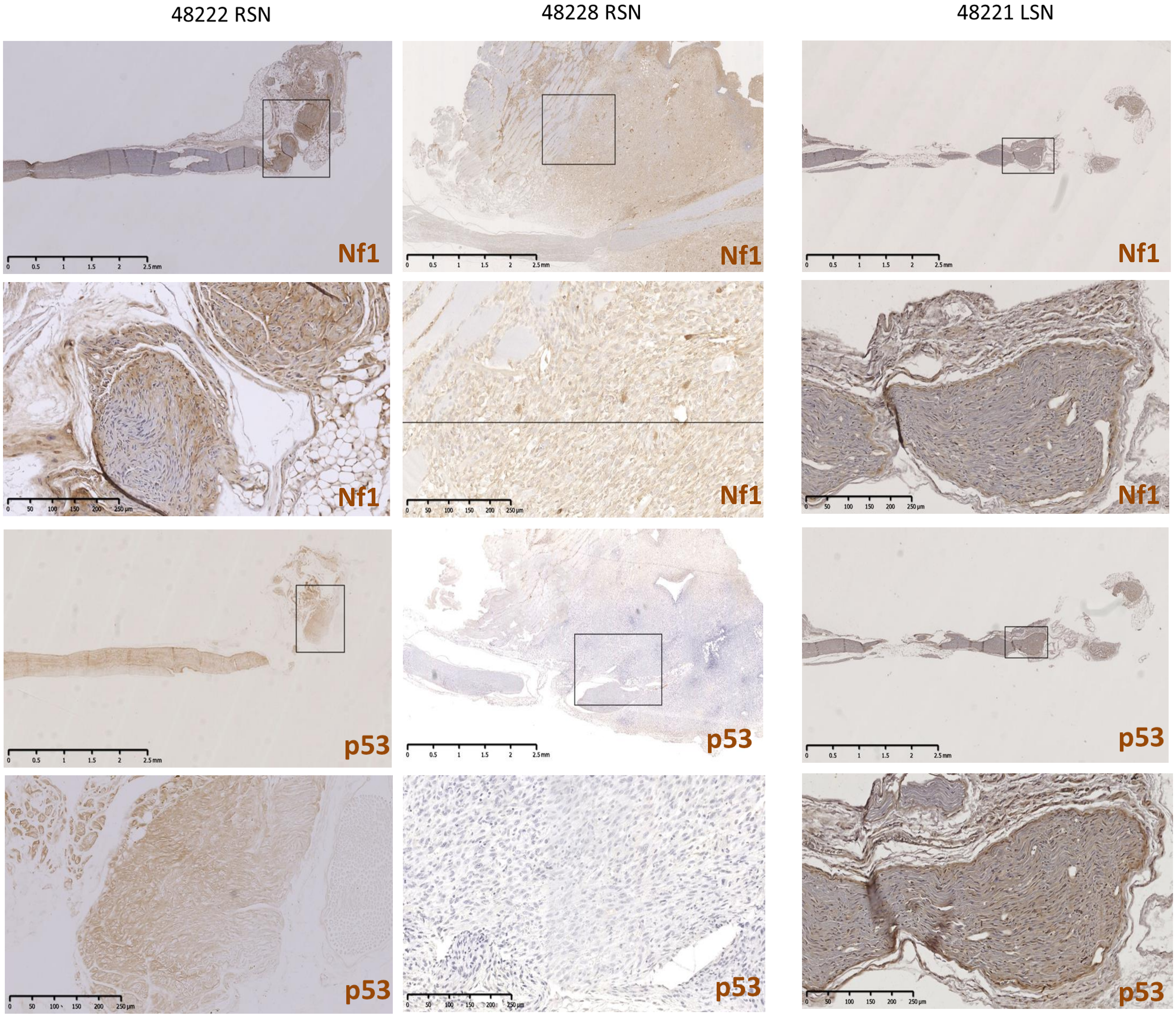

Supplement: S6 Fig — Nf1 and p53 immunostaining of injury-induced sciatic nerve from the NPcis mouse model. (PDF) [file pone.0301040.s006.pdf]
