# Supplementary material for: Revisiting the NPcis mouse model: A new tool to model plexiform neurofibroma
Source: PLoS One. 2024 Jun 20;19(6):e0301040. doi: 10.1371/journal.pone.0301040 (PMC11189233; doi:10.1371/journal.pone.0301040)

S7 Fig

Nf1 and p53 IHC. Spontaneous sarcoma from the NPcis

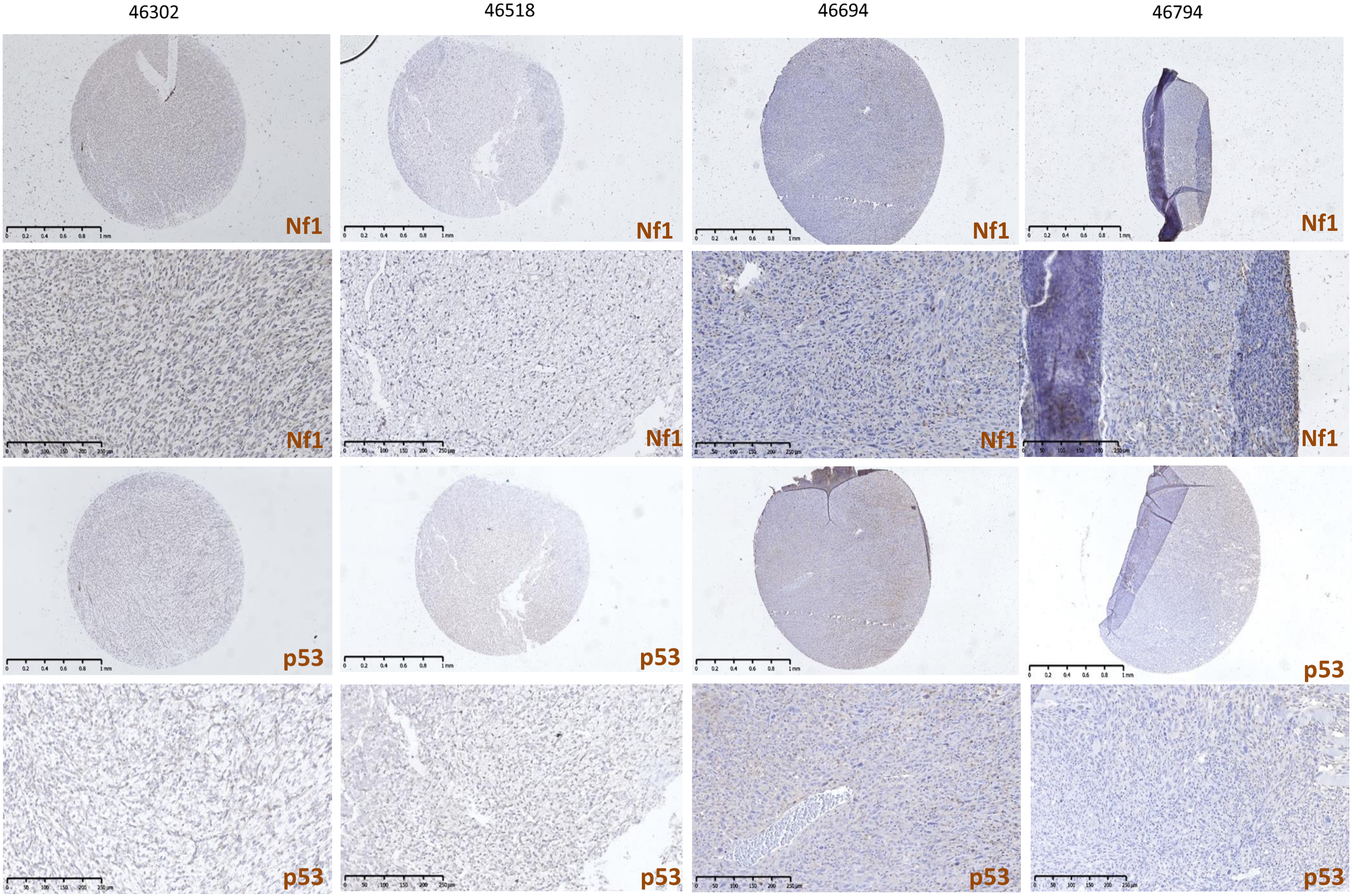

S7 Fig

Nf1 and p53 IHC. Spontaneous sarcoma from the NPcis

46526 46766 46865 46903

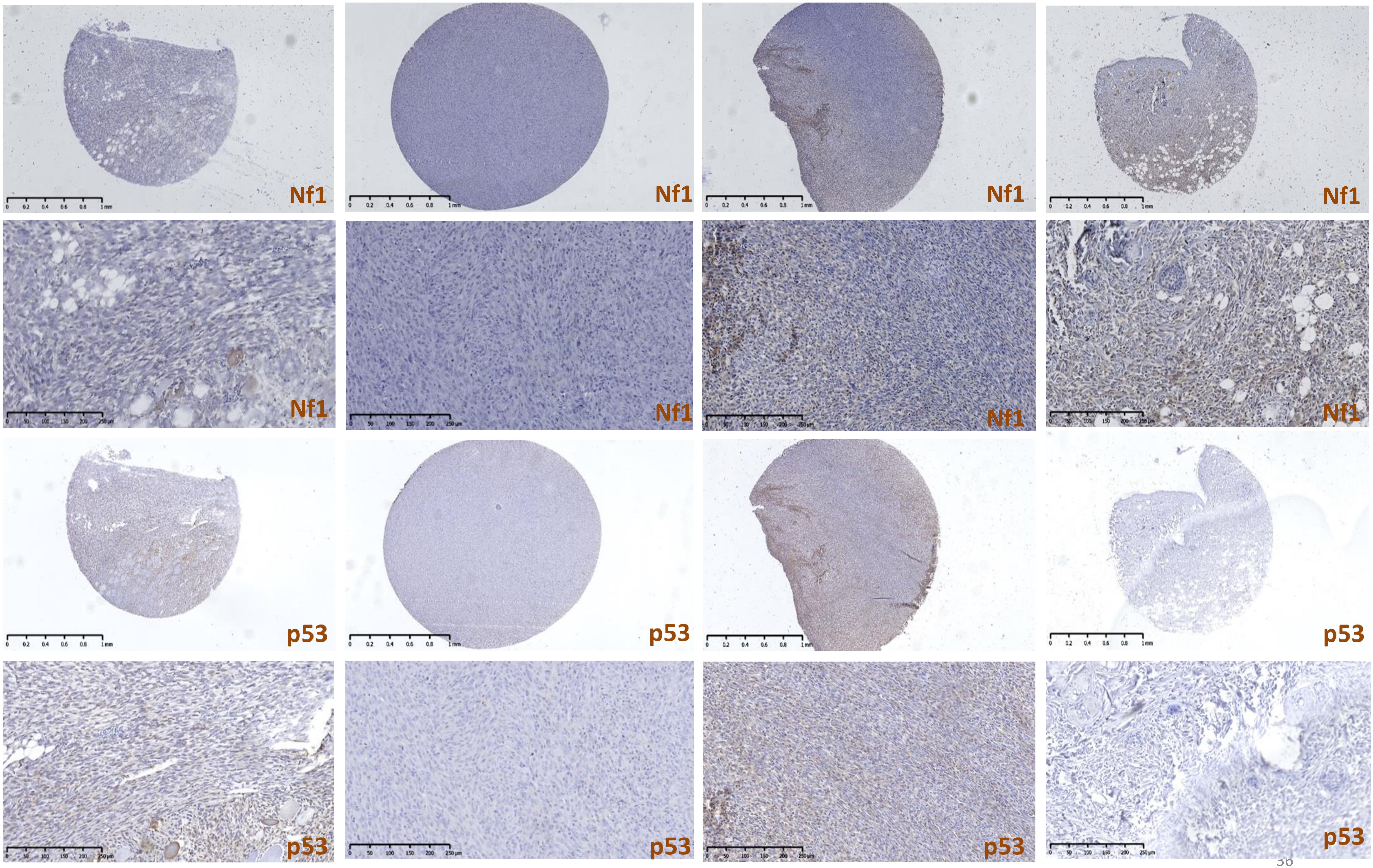

S7 Fig

Nf1 and p53 IHC. Spontaneous sarcoma from the NPcis

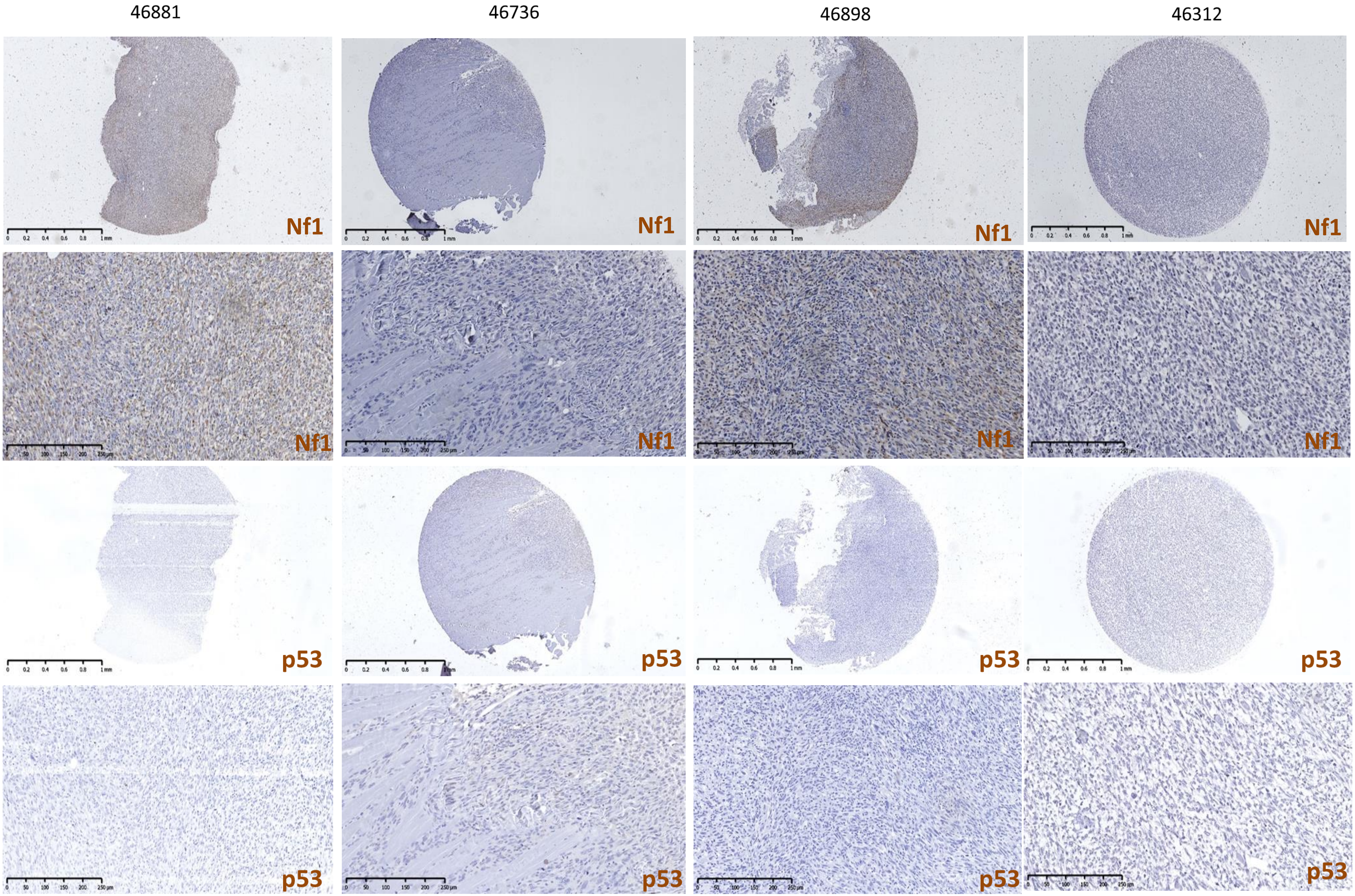

S7 Fig

Nf1 and p53 IHC. Spontaneous sarcoma from the NPcis

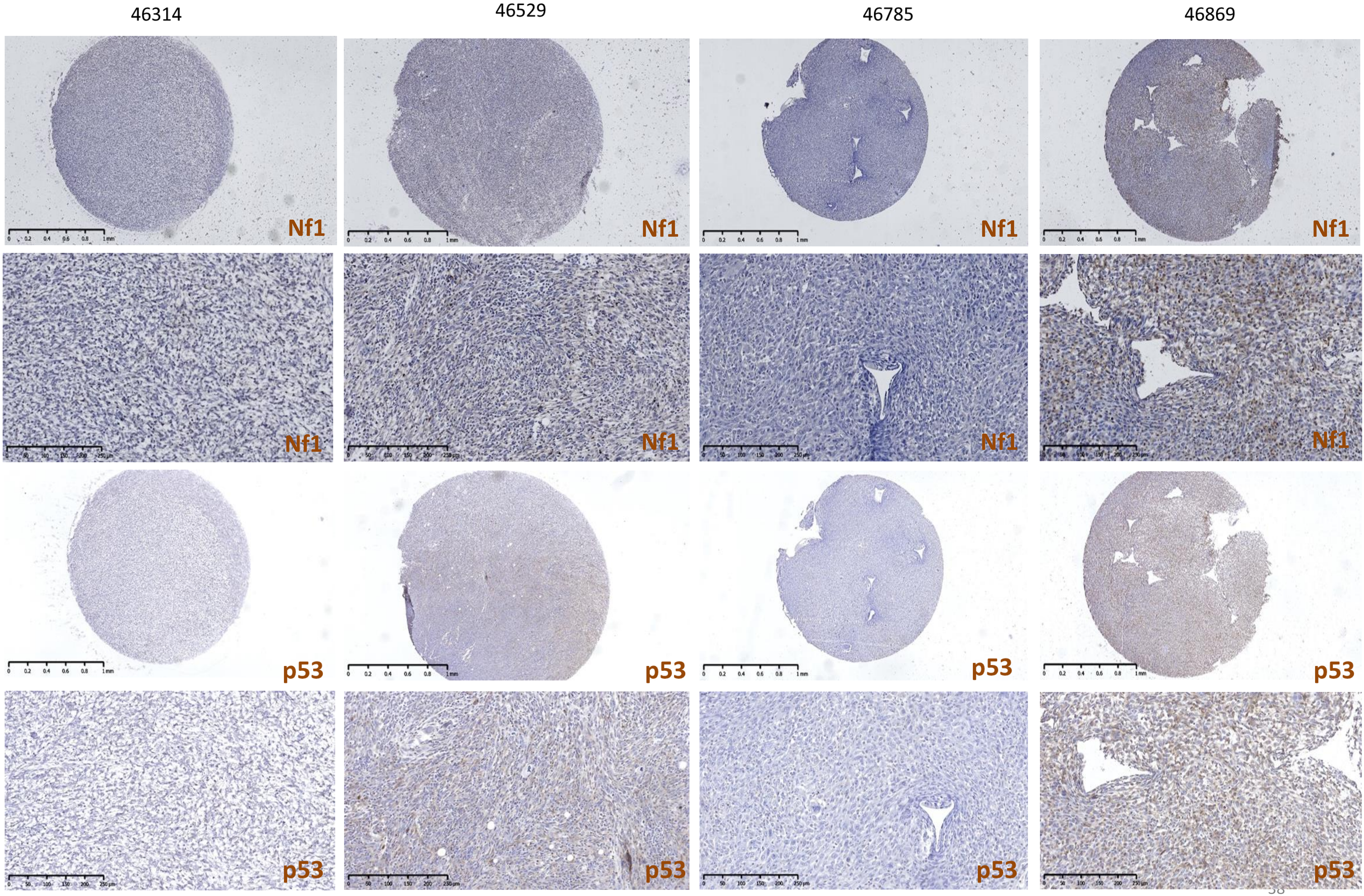

S7 Fig

Nf1 and p53 IHC. Spontaneous sarcoma from the NPcis

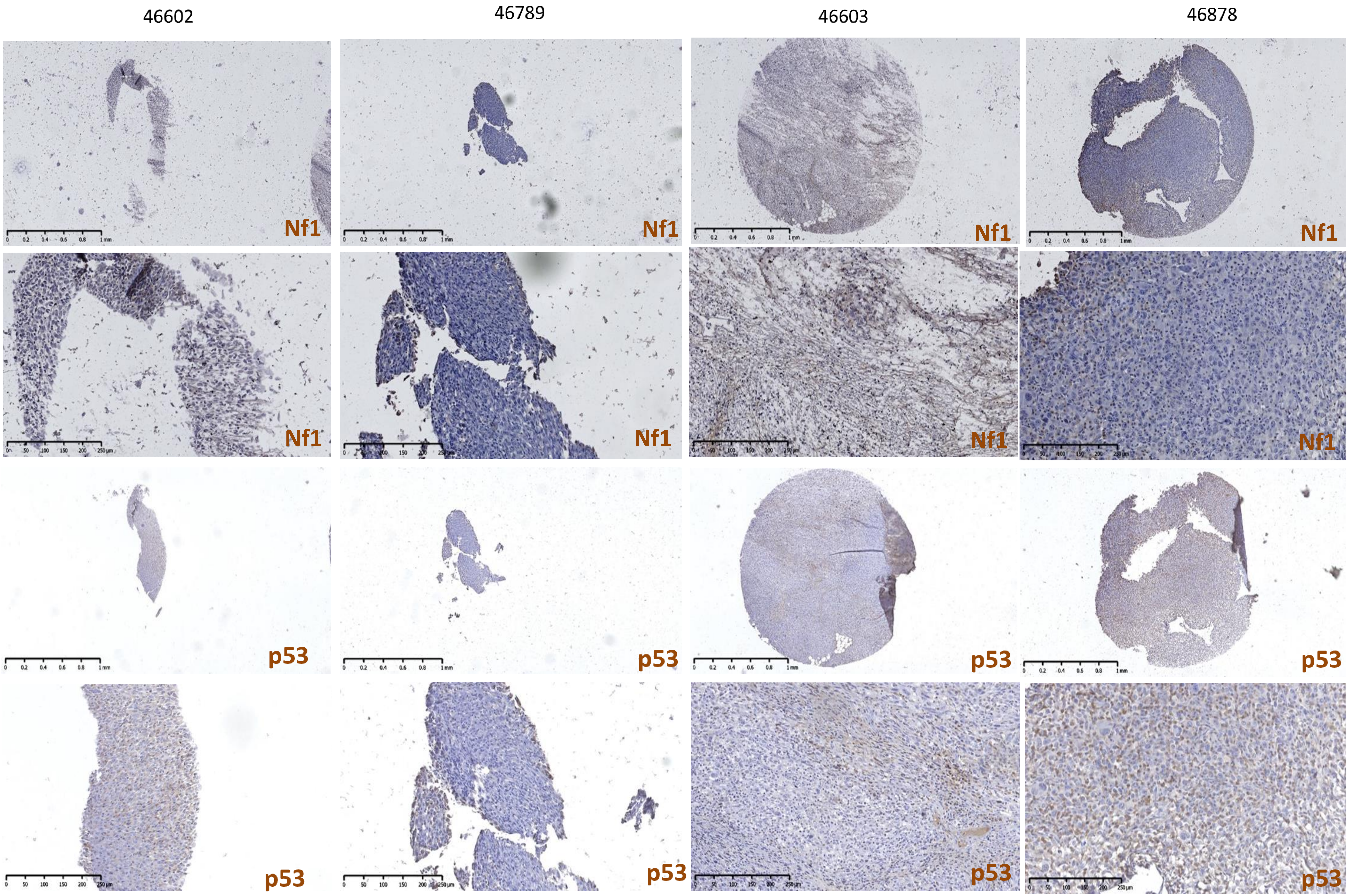

S7 Fig

Nf1 and p53 IHC. Spontaneous sarcoma from the NPcis

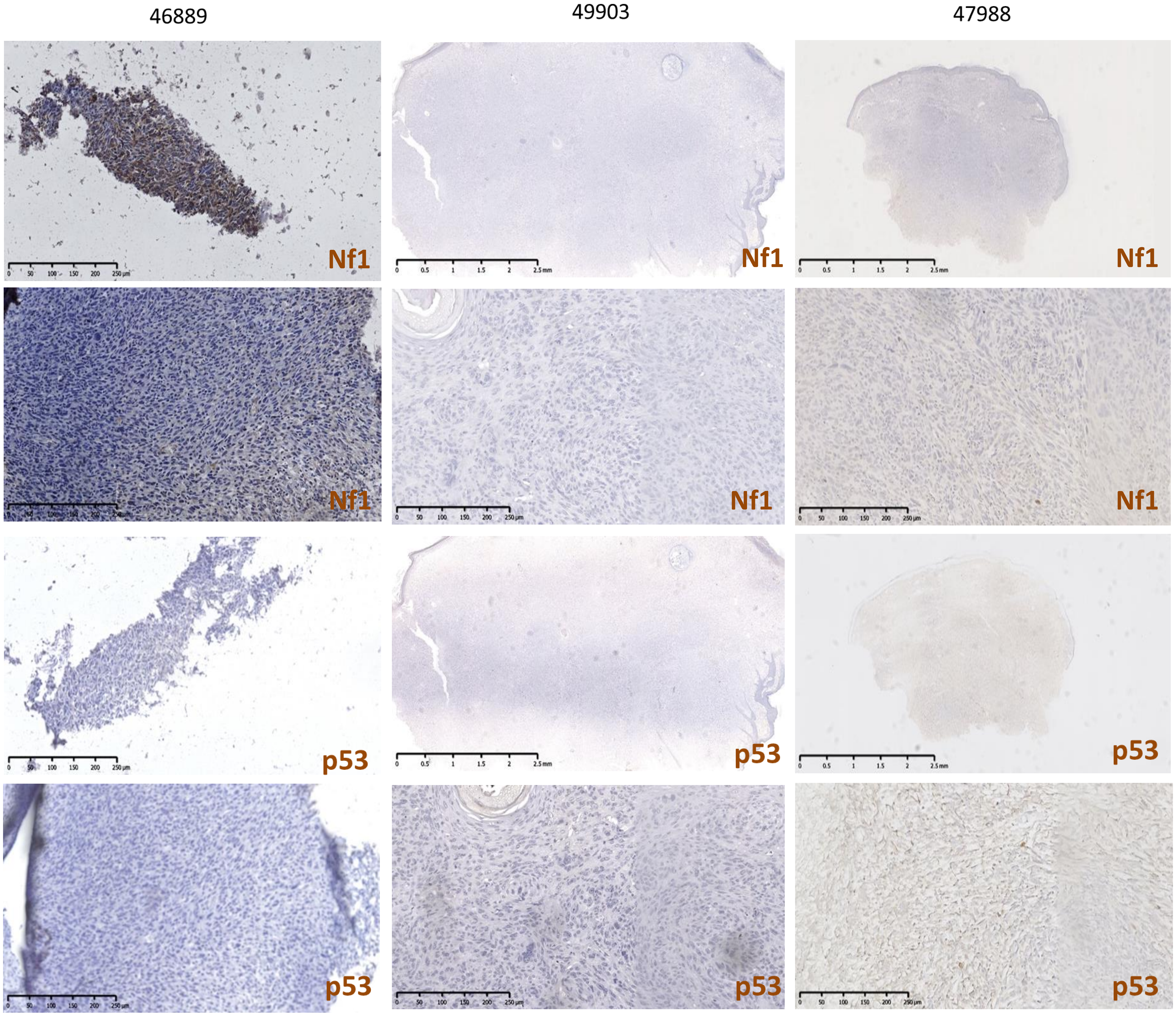

Supplement: S7 Fig — NF1 and p53 immunostaining of spontaneous sarcoma from the NPcis mouse model. (PDF) [file pone.0301040.s007.pdf]
