# Supplementary material for: Revisiting the NPcis mouse model: A new tool to model plexiform neurofibroma
Source: PLoS One. 2024 Jun 20;19(6):e0301040. doi: 10.1371/journal.pone.0301040 (PMC11189233; doi:10.1371/journal.pone.0301040)
Supplement: S1 Table — (PDF) [file pone.0301040.s008.pdf]

S1 Table

Mouse #	**Sex (Fig. 1d)**	**Genotype**	**Mass anatomical location (Fig. 1b)**	**Mass body location (Fig. 1c)**	**Mass histology performed? (Fig. S1)**	**NF clinical signs? (Fig. 2a)**	**Mass at ear tag? (Fig. 5b)**
46519	F	cisNf1p53	N/A	N/A	N/A	Not found	Not found
46863	F	cisNf1p53	N/A	N/A	N/A	Not found	Not found
46869	F	cisNf1p53	N/A	N/A	N/A	Not found	Not found
46794	F	cisNf1p53	N/A	N/A	N/A	Not found	Not found
46303	F	cisNf1p53	N/A	N/A	N/A	Not found	Not found
46310	F	cisNf1p53	N/A	N/A	N/A	Not found	Not found
46502	F	cisNf1p53	N/A	N/A	N/A	Not found	Not found
46517	F	cisNf1p53	N/A	N/A	N/A	Arched back	Not found
46600	F	cisNf1p53	N/A	N/A	N/A	Arched back, low activity	Not found
46696	F	cisNf1p53	N/A	N/A	N/A	Difficulty to deambulate. Arched back	Not found
46734	F	cisNf1p53	N/A	N/A	N/A	thin, low activity	Not found
46513	F	cisNf1p53	N/A	N/A	N/A	thin	Not found
46605	F	cisNf1p53	N/A	N/A	N/A	thin, low activity	Not found
46608	F	cisNf1p53	N/A	N/A	N/A	No	Yes
46740	F	cisNf1p53	N/A	N/A	N/A	cNF	Yes
46768	F	cisNf1p53	N/A	N/A	N/A	Arched back, low activity	Not found
46864	F	cisNf1p53	N/A	N/A	N/A	No	Yes

46870	M	cisNf1p53	N/A	N/A	N/A	Not found	Not found
46886	M	cisNf1p53	N/A	N/A	N/A	Not found	Not found
46901	M	cisNf1p53	N/A	N/A	N/A	Not found	Not found
46902	M	cisNf1p53	N/A	N/A	N/A	Not found	Not found
46314	M	cisNf1p53	N/A	N/A	N/A	Not found	Not found
46316	M	cisNf1p53	N/A	N/A	N/A	Difficulty to deambulate. Arched back	Not found
46602	M	cisNf1p53	N/A	N/A	N/A	No	Not found
46765	M	cisNf1p53	N/A	N/A	N/A	No	Not found
46865	M	cisNf1p53	N/A	N/A	N/A	Arched back, low activity, thin	Not found
46872	F	cisNf1p53	1st mass not recorded; 2nd mass not recorded	1st mass trunk; 2nd mass trunk	No	No	Not found
46899	F	cisNf1p53	1st mass s.c., 2nd mass ear tag	trunk	No	no	Yes
46515	m	cisNf1p53	1st mass s.c., 2nd mass ear tag	trunk	No	No	Yes
46903	M	cisNf1p53	cutaneous	limb	No	No	Not found
46792	M	cisNf1p53	cutaneous	limb	No	No	Not found
46313	M	cisNf1p53	Not recorded	Not recorded	No	No	Not found
46516	M	cisNf1p53	Not recorded	Not recorded	No	No	Not found
46862	F	cisNf1p53	subcutaneous	limb	No	No	Not found
46529	M	cisNf1p53	subcutaneous	trunk	No	No	Not found

46879	m	cisNf1p53	1st mass s.c., 2nd mass ear tag	trunk	Yes	No	Yes
46635	F	cisNf1p53	abdominal	trunk	Yes	no	Not found
46861	F	cisNf1p53	cutaneous	trunk	Yes	No	Not found
46873	M	cisNf1p53	cutaneous	limb	Yes	No	Not found
46909	M	cisNf1p53	cutaneous	trunk	Yes	No	Not found
46567	F	cisNf1p53	Not recorded	Not recorded	Yes	No	Not found
46601	M	cisNf1p53	subcutaneous	limb	Yes	No	Not found
46718	F	cisNf1p53	subcutaneous	trunk	Yes	No	Not found
46882	f	cisNf1p53	subcutaneous	trunk	Yes	No	Not found
46298	F	cisNf1p53	subcutaneous	trunk	Yes	no	Not found
46695	F	cisNf1p53	subcutaneous	trunk	Yes	No	Not found
46736	F	cisNf1p53	subcutaneous	trunk	Yes	no	Not found
46302	M	cisNf1p53	subcutaneous	head & neck	Yes	No	Not found
46312	M	cisNf1p53	subcutaneous	limb	Yes	Difficulty to deambulate	Not found
46518	M	cisNf1p53	subcutaneous	limb	Yes	No	Not found
46526	M	cisNf1p53	subcutaneous	limb	Yes	Difficulty to deambulate	Not found
46766	M	cisNf1p53	subcutaneous	limb	Yes	No	Not found
46881	m	cisNf1p53	subcutaneous	limb	Yes	No	Not found
46603	M	cisNf1p53	subcutaneous	trunk	Yes	No	Not found
46694	M	cisNf1p53	subcutaneous	trunk	Yes	No	Not found
46785	M	cisNf1p53	subcutaneous	trunk	Yes	No	Not found
46789	M	cisNf1p53	subcutaneous	trunk	Yes	No	Not found
46791	M	cisNf1p53	subcutaneous	trunk	Yes	No	Not found
46793	M	cisNf1p53	subcutaneous	trunk	Yes	No	Not found
46878	M	cisNf1p53	subcutaneous	trunk	Yes	No	Not found
46898	M	cisNf1p53	1st mass s.c. ; 2nd mass cutaneous	1st mass trunk; 2nd mass limb	Yes (one)	No	Not found

46296	M	WT	N/A	N/A	N/A	No	Not found
46305	F	WT	N/A	N/A	N/A	No	Not found
46510	M	WT	N/A	N/A	N/A	No	Not found
46566	M	WT	N/A	N/A	N/A	No	Not found
46575	F	WT	N/A	N/A	N/A	No	Not found
46596	M	WT	N/A	N/A	N/A	No	Not found
46599	F	WT	N/A	N/A	N/A	No	Not found
46609	F	WT	N/A	N/A	N/A	No	Not found
46691	M	WT	N/A	N/A	N/A	No	Not found
46692	M	WT	N/A	N/A	N/A	No	Not found
46712	M	WT	N/A	N/A	N/A	No	Not found
46713	M	WT	N/A	N/A	N/A	No	Not found
46714	M	WT	N/A	N/A	N/A	No	Not found
46715	M	WT	N/A	N/A	N/A	No	Not found
46716	F	WT	N/A	N/A	N/A	No	Not found
46717	F	WT	N/A	N/A	N/A	No	Not found
46721	F	WT	N/A	N/A	N/A	No	Not found
46735	M	WT	N/A	N/A	N/A	No	Not found
46737	F	WT	N/A	N/A	N/A	No	Not found
46738	F	WT	N/A	N/A	N/A	No	Not found
46739	F	WT	N/A	N/A	N/A	No	Not found
46741	M	WT	N/A	N/A	N/A	No	Not found
46781	M	WT	N/A	N/A	N/A	No	Not found
46782	M	WT	N/A	N/A	N/A	No	Not found
46783	F	WT	N/A	N/A	N/A	No	Not found
46784	M	WT	N/A	N/A	N/A	No	Not found
46786	F	WT	N/A	N/A	N/A	No	Not found
46787	M	WT	N/A	N/A	N/A	No	Not found
46790	M	WT	N/A	N/A	N/A	No	Not found
46871	F	WT	N/A	N/A	N/A	No	Not found

46880	F	WT	N/A	N/A	N/A	No	Not found
46900	F	WT	N/A	N/A	N/A	No	Not found
46907	M	WT	N/A	N/A	N/A	No	Not found
46914	F	WT	N/A	N/A	N/A	No	Not found
46915	F	WT	N/A	N/A	N/A	No	Not found
46916	F	WT	N/A	N/A	N/A	No	Not found
46295	M	WT	N/A	N/A	N/A	No	Not found
46297	M	WT	N/A	N/A	N/A	No	Not found
46299	M	WT	N/A	N/A	N/A	No	Not found
46304	F	WT	N/A	N/A	N/A	No	Not found
46306	F	WT	N/A	N/A	N/A	No	Not found
46528	M	WT	N/A	N/A	N/A	No	Not found
46581	M	WT	N/A	N/A	N/A	No	Not found
46582	M	WT	N/A	N/A	N/A	No	Not found
46598	M	WT	N/A	N/A	N/A	No	Not found
46604	M	WT	N/A	N/A	N/A	No	Not found
46607	M	WT	N/A	N/A	N/A	No	Not found
46636	M	WT	N/A	N/A	N/A	No	Not found
46638	F	WT	N/A	N/A	N/A	No	Not found
46692	M	WT	N/A	N/A	N/A	No	Not found
46719	F	WT	N/A	N/A	N/A	No	Not found
46720	F	WT	N/A	N/A	N/A	No	Not found
46762	M	WT	N/A	N/A	N/A	No	Not found
46763	M	WT	N/A	N/A	N/A	No	Not found
46906	F	WT	N/A	N/A	N/A	No	Not found
46917	M	WT	N/A	N/A	N/A	No	Not found
