# Supplementary material for: Revisiting the NPcis mouse model: A new tool to model plexiform neurofibroma
Source: PLoS One. 2024 Jun 20;19(6):e0301040. doi: 10.1371/journal.pone.0301040 (PMC11189233; doi:10.1371/journal.pone.0301040)
Supplement: S2 Table — (PDF) [file pone.0301040.s009.pdf]

S2 Table

Mouse #	Genotype	Sex	Damaged method	Lifetime (days)	Reason to euthanize
46533	cisNf1p53	F	needle	120	reach the 3 months point after surgery
46534	cisNf1p53	F	needle	120	reach the 3 months point after surgery
47751	cisNf1p53	M	needle	120	reach the 3 months point after surgery
47622	cisNf1p53	F	needle	48	Mass right flank. Abscess, no sarcoma
47672	cisNf1p53	F	needle	120	reach the 3 months point after surgery
47673	cisNf1p53	F	needle	116	Ambulation difficulties
47674	cisNf1p53	F	needle	113	Thinness
47695	cisNf1p53	M	needle	120	reach the 3 months point after surgery
47696	cisNf1p53	M	needle	120	reach the 3 months point after surgery
47698	cisNf1p53	M	needle	120	reach the 3 months point after surgery
47699	cisNf1p53	M	needle	103	Ambulation difficulties
47702	cisNf1p53	F	needle	120	reach the 3 months point after surgery
47691	WT	F	needle	120	reach the 3 months point after surgery
47694	WT	F	needle	120	reach the 3 months point after surgery
47700	WT	F	needle	120	reach the 3 months point after surgery
47701	WT	F	needle	120	reach the 3 months point after surgery
