# Supplementary material for: Revisiting the NPcis mouse model: A new tool to model plexiform neurofibroma
Source: PLoS One. 2024 Jun 20;19(6):e0301040. doi: 10.1371/journal.pone.0301040 (PMC11189233; doi:10.1371/journal.pone.0301040)
Supplement: S3 Table — (PDF) [file pone.0301040.s010.pdf]

S3 Table

Mouse #	Genotype	Sex	Damaged method	Lifetime (days)	Reason to euthanize
48106	cisNf1p53	M	needle	184	reach 6 months
48107	cisNf1p53	M	needle	184	reach 6 months
48110	cisNf1p53	M	needle	184	reach 6 months
48075	cisNf1p53	M	cut	170	mass lower left limb
48086	cisNf1p53	M	cut	151	N/A
48092	cisNf1p53	M	cut	156	mass upper left limb
48095	cisNf1p53	M	cut	101	mass flank
48099	cisNf1p53	M	cut	67	Found dead
48221	cisNf1p53	M	cut	162	N/A
48222	cisNf1p53	M	cut	162	N/A
48226	cisNf1p53	M	cut	162	Ambulation difficulties
48228	cisNf1p53	M	cut	127	mass lower left limb
