# Supplementary material for: Revisiting the NPcis mouse model: A new tool to model plexiform neurofibroma
Source: PLoS One. 2024 Jun 20;19(6):e0301040. doi: 10.1371/journal.pone.0301040 (PMC11189233; doi:10.1371/journal.pone.0301040)
Supplement: Attachment — Submitted filename: Plante Rebuttal letter v2.pdf [file pone.0301040.s011.pdf]

OBJECT : Resubmission of Plante et al. PONE-D-23-19723

Dear Editors,

Thank you for the constructive comments and opportunity to address the editorial and reviewer`s comments on our manuscript entitled: ``Revisiting the NPcis mouse model: a new tool to model plexiform neurofibroma``. Please find below the point-by-point response.

EDITORIAL COMMENTS

1. Please ensure that your manuscript meets PLOS ONE's style requirements, including those for file naming. The PLOS ONE style templates can be found at https://journals.plos.org/plosone/s/file?id=wjVg/PLOSOne_formatting_sample_main_body.pdf and https://journals.plos.org/plosone/s/file?id=ba62/PLOSOne_formatting_sample_title_authors_affiliations.pdf
We now use the PLoS ONE template

2. Note from Emily Chenette, Editor in Chief of PLOS ONE, and Iain Hrynaszkiewicz, Director of Open Research Solutions at PLOS: Did you know that depositing data in a repository is associated with up to a 25% citation advantage (https://doi.org/10.1371/journal.pone.0230416)? If you've not already done so, consider depositing your raw data in a repository to ensure your work is read, appreciated and cited by the largest possible audience. You'll also earn an Accessible Data icon on your published paper if you deposit your data in any participating repository (https://plos.org/open-science/open-data/#accessible-data).
Thank you for the suggestion.

3. Thank you for stating the following financial disclosure:
"Fonds de recherche du Québec - Santé. Grant # 281660"
Please state what role the funders took in the study. If the funders had no role, please state: ""The funders had no role in study design, data collection and analysis, decision to publish, or preparation of the manuscript.""
If this statement is not correct you must amend it as needed.
Please include this amended Role of Funder statement in your cover letter; we will change the online submission form on your behalf.
The funder had no role, therefore thank you for amending the funder role in the online submission form as ""The funders had no role in study design, data collection and analysis, decision to publish, or preparation of the manuscript.""

4. Please expand the acronym “FRQS” (as indicated in your financial disclosure) so that it states the name of your funders in full. This information should be included in your cover letter; we will change the online submission form on your behalf.
FRQS stands for ``Fonds de Recherche du Québec – Santé``. Thank you for amending it in the online submission form.

5. Your ethics statement should only appear in the Methods section of your manuscript. If your ethics statement is written in any section besides the Methods, please move it to the Methods section and delete it from any other section. Please ensure that your ethics statement is included in your manuscript, as the ethics statement entered into the online submission form will not be published alongside your manuscript.
We moved any ethic statement to the Method section.

6. Please include captions for your Supporting Information files at the end of your manuscript, and update any in-text citations to match accordingly. Please see our Supporting Information guidelines for more information: http://journals.plos.org/plosone/s/supporting-information.
We now follow the PLoS ONE template. The list of Supporting Information is now found at the end of the manuscript.

7. Please review your reference list to ensure that it is complete and correct. If you have cited papers that have been retracted, please include the rationale for doing so in the manuscript text, or remove these references and replace them with relevant current references. Any changes to the reference list should be mentioned in the rebuttal letter that accompanies your revised manuscript. If you need to cite a retracted article, indicate the article’s retracted status in the References list and also include a citation and full reference for the retraction notice.
We fully revised the reference list.

REVIEWERS` COMMENTS

Reviewer #1: Because the existing mouse models of neurofibroma and MPNST do not recapitulate the key clinical and molecular features of the corresponding human tumors, the authors aimed to establish a model that recapitulated the human disease clinically and molecularly. Therefore, they revisited the NP cis model. The paper is interesting, well written and clear. The Authors also discussed the current state of NF mouse models, highlighting model relationship to human tumors.
We thank the reviewer for the enthusiasm about our manuscript.

Reviewer #2: In this manuscript Plante et al re-characterized the CisNf1+/-;P53+/- (NPcis) mouse. Consistent with the published data, the NPcis mouse developed malignant peripheral nerve sheath tumor (GEM-PNST). The authors also observed lesions reminiscent of a neurofibroma in a peripheral nerve, a cutaneous neurofibroma, and para-spinal neurofibroma on rare occasions in NPcis mice by a systematic histological analysis. They also observed that 10% of the mice developed GEM-PNSTs spontaneously. More interestingly, about half of the sciatic nerves from NPcis mice developed plexiform neurofibromas (PNFs) within 1-6 months when intentionally injured. Thus, this might provide a procedure to turn the widely used NPcis sarcoma model into a model recapitulating PNF. The authors have provided extensive of histology/immunohistochemistry data. Overall, this is a very well-organized manuscript. Most of the data are convincing. However, some of the following concerns diminished my enthusiasm for publication at current version:
Again, we thank the reviewer for the enthusiasm about our manuscript.

1. Nf-/- Schwann cells are the pathological cells for PNFs. In the NPcis nerve injury model, is there loss of heterozygosity (LOH)? Is there any difference between the mice with PNF and without PNF in the level of LOH? What about the environment change, especially the macrophages.

All excellent points. Ideally, we would have performed DNA sequencing. Unfortunately, the only material output from this study is FFPE slides.

-To determine whether or not there is LOH, we did the following two experiments which are featured in a brand-new Fig. 7 along with S6 and S7 Fig:

a) **We performed immunostaining using NF1 and p53 antibodies on normal sciatic nerves (no injury) from wild-type mice (n=8), injury-induced sciatic nerves from NPcis mice that develop pNF (n=12), injury-induced sciatic nerves from NPcis mice that did not develop (n=9) pNF and spontaneous sarcoma from NPcis mice (n=23) "Fig 7a". Then, the staining intensity was scored and output as a bar graph representing the percentage of tissues assigned to each staining intensity level. The results indicate that NF1 and p53 expression are largely preserved in non-malignant tissues whereas it is lowly expressed in malignant tissues as expected "Fig 7b".**

b) **We also performed qPCR using *Nf1* and *p53* specific primers on normal sciatic nerves (no injury) from wild-type mice (n=2), injury-induced sciatic nerves from NPcis mice that develop pNF (n=2), injury-induced sciatic nerves from NPcis mice that did not develop (n=2) pNF and spontaneous sarcoma from NPcis mice (n=2). Again, the results indicate that *Nf1* and *p53* expression are largely preserved in non-malignant tissues. More precisely, Nf1 expression is not significantly different between NPcis nerves and pNF but it is for p53. As expected, both Nf1 and p53 expression is significantly decreased in sarcoma compared to normal nerve from NPcis mice "Fig 7c". Thus, it suggests that loss of heterozygosity of *Nf1* and *p53* did not took place in injury-induced pNF although alternative strategies to lower p53 expression may exist.**

-To determine any changes in macrophages, we performed IHC using Iba1 antibodies on injury-induced sciatic nerves from NPcis mice that develop pNF (n=7), injury-induced sciatic nerves from NPcis mice that did not develop (n=8) pNF. As expected we found positive staining in the injury-induced pNF and minimal staining in the injury-induced sciatic nerves that did not develop pNF. This new result is now presented as S5 Fig.

2. Is there any histological difference on PNFs developed by longitudinal or cross section procedure?

We revisited the H&E from injury-induced neurofibroma from the ``needle`` and the ``cut`` to evaluate any difference in the histologies. This comparison is now shown in S4 Fig.

3. Some of the NPcis mice also develop brain tumors. This can cause mouse death too. It is important to be included in the characteristics.

It is true that NPcis develop a plethora of manifestations, including brain tumors. However, we strictly focused on the benign manifestations of the skin (cNF) and peripheral nervous system (pNF) in this study.
